# Psychiatric symptoms caused by cannabis constituents: a systematic review and meta-analysis

**DOI:** 10.1016/S2215-0366(20)30074-2

**Published:** 2020-04

**Authors:** Guy Hindley, Katherine Beck, Faith Borgan, Cedric E Ginestet, Robert McCutcheon, Daniel Kleinloog, Suhas Ganesh, Rajiv Radhakrishnan, Deepak Cyril D'Souza, Oliver D Howes

**Affiliations:** a Department of Psychosis Studies, Institute of Psychiatry, Psychology and Neuroscience, King's College London, London, UK; b Department of Biostatistics and Health Informatics, Institute of Psychiatry, Psychology and Neuroscience, King's College London, London, UK; c South London and the Maudsley NHS Foundation Trust, London, UK; d MRC London Institute of Medical Sciences, Hammersmith Hospital Campus, London, UK; e Institute of Clinical Sciences, Faculty of Medicine, Imperial College London, London, UK; f Department of Intensive Care Medicine, Leiden University Medical Hospital, Leiden, Netherlands; g Department of Psychiatry, Yale University School of Medicine, New Haven, CT, USA; h Abraham Ribicoff Research Facilities, Connecticut Mental Health Center, New Haven, CT, USA; i VA Connecticut Healthcare System, West Haven, CT, USA

## Abstract

**Background:**

Approximately 188 million people use cannabis yearly worldwide, and it has recently been legalised in 11 US states, Canada, and Uruguay for recreational use. The potential for increased cannabis use highlights the need to better understand its risks, including the acute induction of psychotic and other psychiatric symptoms. We aimed to investigate the effect of the cannabis constituent Δ^9^-tetrahydrocannabinol (THC) alone and in combination with cannabidiol (CBD) compared with placebo on psychiatric symptoms in healthy people.

**Methods:**

In this systematic review and meta-analysis, we searched MEDLINE, Embase, and PsycINFO for studies published in English between database inception and May 21, 2019, with a within-person, crossover design. Inclusion criteria were studies reporting symptoms using psychiatric scales (the Brief Psychiatric Rating Scale [BPRS] and the Positive and Negative Syndrome Scale [PANSS]) following the acute administration of intravenous, oral, or nasal THC, CBD, and placebo in healthy participants, and presenting data that allowed calculation of standardised mean change (SMC) scores for positive (including delusions and hallucinations), negative (such as blunted affect and amotivation), and general (including depression and anxiety) symptoms. We did a random-effects meta-analysis to assess the main outcomes of the effect sizes for total, positive, and negative PANSS and BPRS scores measured in healthy participants following THC administration versus placebo. Because the number of studies to do a meta-analysis on CBD's moderating effects was insufficient, this outcome was only systematically reviewed. This study is registered with PROSPERO, CRD42019136674.

**Findings:**

15 eligible studies involving the acute administration of THC and four studies on CBD plus THC administration were identified. Compared with placebo, THC significantly increased total symptom severity with a large effect size (assessed in nine studies, with ten independent samples, involving 196 participants: SMC 1·10 [95% CI 0·92–1·28], p<0·0001); positive symptom severity (assessed in 14 studies, with 15 independent samples, involving 324 participants: SMC 0·91 [95% CI 0·68–1·14], p<0·0001); and negative symptom severity with a large effect size (assessed in 12 studies, with 13 independent samples, involving 267 participants: SMC 0·78 [95% CI 0·59–0·97], p<0·0001). In the systematic review, of the four studies evaluating CBD's effects on THC-induced symptoms, only one identified a significant reduction in symptoms.

**Interpretation:**

A single THC administration induces psychotic, negative, and other psychiatric symptoms with large effect sizes. There is no consistent evidence that CBD induces symptoms or moderates the effects of THC. These findings highlight the potential risks associated with the use of cannabis and other cannabinoids that contain THC for recreational or therapeutic purposes.

**Funding:**

UK Medical Research Council, Maudsley Charity, Brain and Behavior Research Foundation, Wellcome Trust, and the UK National Institute for Health Research.

## Introduction

Cannabis is one of the most widely used psychoactive substances worldwide, with 6–7% of the population in Europe and 15·3% of the population in the USA using it each year.^1^ There is a global trend towards decriminalisation and legalisation,^1^ with 11 US states, Canada, and Uruguay now permitting the sale and recreational use of cannabis in addition to its medicinal use.^1^ Given the projected increase in rates of cannabis use,^2^ the increasing potency of cannabis and cannabis-based products, and the burgeoning interest in the therapeutic potential of cannabinoids,^3^ it is timely to assess the psychiatric effects of cannabis constituents.

J J Moreau^4^ first described an association between cannabis use and psychotic symptoms, such as paranoia and hallucinations, more than 150 years ago. Subsequently, the main psychoactive constituent of cannabis, Δ^9^-tetrahydrocannabinol (THC), was shown to induce a significant increase in psychotic (also referred to as positive) symptoms as well as negative symptoms, such as poor rapport, and general psychiatric symptoms, such as depression, relative to placebo.^5^ Multiple independent studies have explored the psychotomimetic properties of THC since.^5^, ^6^, ^7^, ^8^, ^9^, ^10^, ^11^, ^12^, ^13^, ^14^, ^15^, ^16^, ^17^, ^18^ Although most of these studies support the original findings, discrepancies exist,^7^, ^13^, ^18^ highlighting the need to determine the consistency and magnitude of these effects. Furthermore, potential modifiers of these effects, such as dose, previous cannabis use, route of administration, age, sex, tobacco use, and type of THC, have not been systematically evaluated.

**Research in context**

**Evidence before this study**

Studies in healthy people indicate that the cannabis constituent Δ^9^-tetrahydrocannabinol (THC) can induce positive and negative symptoms but findings have been inconsistent. Thus, the magnitude, consistency, and moderators of the induction of schizophreniform and other symptoms by THC remain unclear, including the role of other cannabis constituents such as cannabidiol (CBD). MEDLINE (from Jan 1, 1946, to May 21, 2019), Embase (from Jan 1, 1974, to May 21, 2019), and PsycINFO (from Jan 1, 1806, to May 21, 2019) were searched using the following keywords: (“THC” OR “tetrahydrocannabinol” OR “9THC” OR “9tetrahydrocannabinol” OR “delta9THC” OR “d9THC” OR “delta9tetrahydrocannabinol” OR “dronabinol” OR “marinol” OR “bedrobinol” OR “anandamide” OR “methanandamide” OR “WIN,55,212-2” OR “ACPA” OR “CP55940” OR “bedrocan” OR “spice” OR “JWH-018” OR “AM251” OR “SR161716A” OR “rimonabant” OR “cannabidiol” OR “CBD” OR “cannabinoid”) AND (“BPRS” OR “brief psychiatric rating scale” OR “PANSS” OR “positive and negative syndrome scale”).

**Added value of this study**

In this meta-analysis of 15 studies, we determined that the acute administration of THC induces positive, negative, and other symptoms associated with schizophrenia and other mental disorders in healthy adults with large effect sizes. Evidence of CBD's modifying effect is inconclusive. We also found lower induction of psychotic symptoms by THC in studies with more tobacco smokers, and that cannabis use did not moderate the induction of symptoms by THC. These findings extend the literature by systematically showing that THC induces psychotic and other psychiatric symptoms across a range of forms, routes of administration, doses, and settings.

**Implications of all the available evidence**

Our finding that THC induces positive and other psychiatric symptoms highlights the risks associated with the use of cannabis products, which should be factored into risk–benefit discussions between patients and medical practitioners. This work will inform regulators, public health initiatives, and policy makers considering the medical use of cannabis products or their legalisation for recreational use. Our findings also have implications for mental health policy in terms of education on risks and harm minimisation strategies for products containing THC, and for research into effects in people who might be vulnerable to mental illness.

There is increasing interest in the effects of cannabidiol (CBD), another constituent of cannabis.^19^ CBD does not induce schizophreniform symptoms itself.^7^, ^11^, ^20^ Cannabis containing higher proportions of CBD has been associated with fewer subclinical psychotic symptoms in people who use cannabis recreationally in naturalistic studies.^21^, ^22^ This finding has led to suggestions that CBD has antipsychotic properties, with some promising results in people with schizophrenia.^23^, ^24^ However, results from controlled studies evaluating whether CBD can attenuate THC-induced psychiatric symptoms are mixed.^7^, ^20^, ^25^, ^26^ As the THC-to-CBD ratio of street cannabis continues to increase,^27^ clarification of the moderating effects of CBD is needed.

We aimed to investigate the psychotomimetic effects of THC and CBD alone and in combination on healthy volunteers to determine the magnitude and consistency of the psychiatric effects of THC and CBD, to investigate the moderating effects of CBD on THC-induced symptoms, and to evaluate the moderating effects of demographic and clinical factors on the induction of symptoms.

## Methods

### Search strategy and selection criteria

For this systematic review and meta-analysis, inclusion criteria were double-blind studies that included healthy participants; reported symptom changes in response to acute administration of intravenous, oral, or inhaled THC or CBD; contained either a placebo condition (for the effects of THC or CBD alone) or concurrent administration of THC plus CBD or placebo CBD (for the moderation of THC effects by CBD); used a within-person, crossover design; reported total, positive, or negative symptoms using BPRS or PANSS; and presented data allowing the calculation of the standardised mean difference and deviation between the THC and placebo condition.

Exclusion criteria were studies not involving a control condition, using an active control, or administering concurrent medication (besides CBD for the systematic review of CBD plus THC); studies with absence of measures in either the THC or control condition; studies not written in English; studies not reporting original data; studies only providing p or *t* values, change measurements, or effect sizes; studies with two or fewer participants in each group; and studies involving concurrent administration of other pharmacological compounds.

To ensure comparable and reliable outcome measures, we focused on studies that used standardised, well validated rating scales of psychotic, negative and general psychiatric symptoms (the Brief Psychiatric Rating Scale [BPRS] and the Positive and Negative Syndrome Scale [PANSS]).^28^, ^29^ These tools are designed to measure change in symptoms across psychopathological symptom domains relevant to schizophrenia, including positive (psychotic-like) symptoms such as hallucinations, delusions, and thought disorder, as well as negative symptoms such as blunted affect, anhedonia and amotivation, and general psychopathology, including depressive, cognitive, and anxiety symptoms. Additional searches were made for other well validated scales (Scale for the Assessment of Negative Symptoms, Scale for the Assessment of Positive Symptoms, and Community Assessment of Psychic Experience), details of which can be found in the (appendix p 2). These searches were not in the original protocol and were done at the request of reviewers.

Two authors (GH and KB) independently did the search and data extraction (appendix p 2). MEDLINE (from Jan 1, 1946, to May 21, 2019), Embase (from Jan 1, 1974, to May 21, 2019), and PsycINFO (from Jan 1, 1806, to May 21, 2019) were searched. The following keywords were used: (“THC” OR “tetrahydrocannabinol” OR “9THC” OR “9tetrahydrocannabinol” OR “delta9THC” OR “d9THC” OR “delta9tetrahydrocannabinol” OR “dronabinol” OR “marinol” OR “bedrobinol” OR “anandamide” OR “methanandamide” OR “WIN,55,212-2” OR “ACPA” OR “CP55940” OR “bedrocan” OR “spice” OR “JWH-018” OR “AM251” OR “SR161716A” OR “rimonabant” OR “cannabidiol” OR “CBD” OR “cannabinoid”) AND (“BPRS” OR “brief psychiatric rating scale” OR “PANSS” OR “positive and negative syndrome scale”). Meta-analyses, review articles, and included manuscripts were hand-searched for missing studies. Abstracts were screened and the full texts of suitable studies were obtained. If studies used BPRS or PANSS, but data for any of three scales (total, negative, or positive) or additional variables of interest were missing, the authors were contacted for data. Two authors (GH and KB) selected the final studies included in the systematic review and meta-analysis. Conflicts were resolved by discussion between these two authors and ODH where necessary. We contacted study authors to confirm that studies had independent samples. We did the meta-analysis according to the Meta-analysis of Observational Studies in Epidemiology (MOOSE) framework.^30^ The protocol is available online .

### Data analysis

GH and KB independently extracted data from studies. Data extraction was cross-checked to ensure accuracy. Where there were discrepancies, these were resolved by discussion with ODH. The main outcome measures were the effect sizes for total, positive, and negative PANSS and BPRS scores measured in healthy participants following THC administration versus placebo.

If more than one dose or timepoint was reported, the data for the maximum dose or the timepoint associated with the highest mean symptom score for the THC condition with the corresponding placebo score were extracted because we aimed to determine the maximum possible effect. Variables extracted were author, year of publication, number of participants, mean age, proportion of males, proportion of current tobacco smokers, mean total lifetime cannabis exposures, details of control condition and randomisation procedure, inclusion and exclusion criteria, route and dose of THC, symptom measure used and subscales reported, timing of measure relative to administration of THC, and mean and SD of symptom scales. If dose was presented as mg/kg, the mean dose delivered was calculated by multiplying the dose per kg by the mean weight in kg of participants.

We assessed risk of bias using the Newcastle-Ottawa Scale.^31^ Disagreements were resolved by discussion between GH, KB, and ODH. Studies with scores of 7 or more were considered to have low risk of bias.^32^ If duplicate data were suspected, the authors were contacted for confirmation and the study with the largest sample size or the largest number of required variables was chosen, with sample size taking precedence.

We used random-effects models based on restricted maximum likelihood estimation in all analyses, since between-study heterogeneity was expected because of the variability in experimental methods and sample characteristics. Given that we were examining within-person studies, the SMC was calculated as a measure of the magnitude of placebo–THC differences. We also calculated the 95% CI of the SMC. The SMC was defined for each study as follows:

$$\frac{M_{\text{THC}}-M_{\text{pla}}}{\sqrt{\left({\text{SD}}_{\text{THC}}^{2}+{\text{SD}}_{\text{Pla}}^{2}-2{\text{rSD}}_{\text{THC}}{\text{SD}}_{\text{Pla}}\right)}}$$

where *M*_THC_ and *M*_Pla_ are the mean scores for the THC and placebo conditions, respectively, and SD_THC_, and SD_Pla_ are the standard deviations for the THC and placebo conditions, respectively; *r* denotes the between-condition correlation for symptom scores under the THC and placebo conditions. An SMC value of less than 0·40 was considered a small effect, 0·40–0·70 a moderate effect, and more than 0·70 a large effect.^33^ The correlation coefficient (*r*) was set to 0·5 for all studies in our main analysis on the basis of previous literature.^34^ We did a sensitivity analysis to evaluate the influence of this assumption on our main results by refitting our model using *r* values of 0·1 and 0·7 (appendix p 20).

We assessed inconsistency across studies using the Cochran Q statistic and the *I*^2^ statistic.^35^, ^36^ An *I*^2^ value of less than 25% was considered to have low inconsistency, 25% to 75% indicated medium inconsistency, and greater than 75% indicated high inconsistency.^36^, ^37^ We also did leave-one-out sensitivity analyses. Publication bias and selective reporting were assessed using Egger's regression test^38^ and represented diagrammatically with funnel plots. If missing studies were identified, they were imputed using trim-and-fill analyses. We did meta-regression and subgroup analyses to evaluate the potential modifying effect of age (mean), sex (proportion of male participants), proportion of tobacco smokers, dose (mg), current cannabis use (studies in which participants' recent use was confirmed with a positive urine drug screen for cannabis *vs* studies in which participants had confirmed abstinence from recent cannabis with a negative urine drug screen for cannabis at screening),^39^ frequency of cannabis use (mean total exposures >100 *vs* mean total exposures <100),^39^ route of administration (oral *vs* inhaled *vs* intravenous),^40^ type of THC (purified *vs* synthetic), symptom scale (BPRS *vs* PANSS), study quality (Newcastle-Ottawa Scale score), and study author (D'Souza group *vs* other).^41^ Finally, we did an exploratory analysis comparing the magnitude of the effect of THC effects on positive, negative, and general symptoms (see appendix p 2 for further details). Because of the range of timepoints reported by each study and the variation in half-life according to route of administration, we were unable to meta-analyse duration of symptoms. Given the clinical relevance of this issue, we summarise the findings of the included studies in the appendix (pp 3, 20).

The significance level for all statistical tests was p<0·05 (two tailed). All raw data are provided in the appendix (pp 4–7). All code used in the analysis can be requested from the corresponding author. Statistical analyses were done with the metafor package (version 1.9-9) in the statistical programming language R (version 3.3.1).

This study is registered with PROSPERO, CRD42019136674.

### Role of the funding source

The funders of the study had no role in study design, data collection, data analysis, data interpretation, or writing of the report. The corresponding author had full access to all the data in the study and had final responsibility for the decision to submit for publication.

## Results

**Figure 1:**
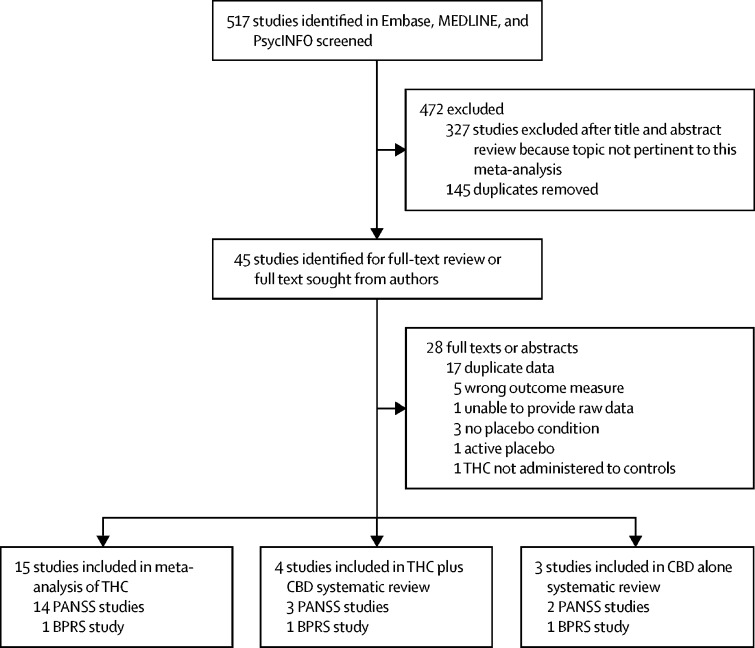
Study selection BPRS=Brief Psychiatric Rating Scale. CBD=cannabidiol. PANSS=Positive and Negative Syndrome Scale. THC=Δ^9^-tetrahydrocannabinol.

Of 517 studies screened, 15 studies met the inclusion criteria for meta-analysis of the acute administration of THC in healthy individuals (figure 1). Four studies on CBD's effect on THC were identified, which was insufficient for a meta-analysis; therefore, only a systematic review was done. Table 1 provides summary details of the studies included, with further details provided in the appendix (p 8). 331 healthy controls received both THC and placebo conditions (see table 1 for summary of placebos used). Regarding study quality, 13 of 15 studies had scores of 7 or more on the Newcastle Ottawa Scale, indicating low risk of bias.^5^, ^6^, ^8^, ^9^, ^10^, ^12^, ^13^, ^14^, ^15^, ^16^, ^17^, ^18^, ^44^ Two studies had scores of 6, implying moderate risk of bias.^7^, ^11^ The most common limitation was a non-representative sample (appendix p 10). Studies were confirmed to be independent for 13 of the studies^5^, ^6^, ^7^, ^8^, ^9^, ^10^, ^12^, ^13^, ^14^, ^15^, ^16^, ^17^, ^18^, ^44^ included. For two studies,^11^, ^12^ we were unable to contact the authors. As the study descriptions do not indicate sample overlap, we included them in the main analysis. However, in case there was overlap, we repeated the main analysis excluding the smaller study.^11^ This analysis demonstrated that the main findings were essentially the same (appendix p 20).

**Table 1:** Within-person study samples and designs involving healthy individuals receiving THC and placebo

	**Sample size**	**Mean age (SD), years**	**Male: female**	**Randomised order**	**Route**	**Dose**	**Placebo condition**	**Scale and subscales**	**Time between dose and measurement**
Barkus et al (2011)^6^	9	26·3 (4·2)	9:0	Yes	Intravenous	2·5 mg	2·5% ethanol plus saline	PANSS: positive and negative	30 min
Bhattacharyya et al (2015)^12^	36	26·0 (5·6)	36:0	Yes	Oral	10 mg	Matched placebo–capsule	PANSS: total and positive	120 min
D'Souza et al (2012)^16^	26	25·9 (7·8)	17:9	Yes	Intravenous	0·03 mg/kg	Ethanol*	PANSS: total, positive, and negative	10 mins
D'Souza et al (2004)^5^	18	29	10:5	Yes	Intravenous	5 mg	Ethanol*	PANSS: positive and negative	10 min positive; 80 min negative
D'Souza et al (2008)^14^	20	24·9 (7·0)	14:6	Yes	Intravenous	5 mg	Ethanol*	PANSS: total, positive, and negative	15 min
D'Souza et al (2009; low cannabis use sample)^15^†	14	25·9 (8·0)	11:3	No	Intravenous	0·0286 mg/kg	Matched vehicle*	PANSS: positive	15 min
D'Souza et al (2009; high cannabis use sample)^15^†	9	22·7 (2·8)	9:0	No	Intravenous	0·0286 mg/kg	Matched vehicle*	PANSS: positive	15 min
Morrison et al (2009)^8^	21	28 (6)	21:0	Yes	Intravenous	2·5 mg	Normal saline	PANSS: positive	30 min
Morrison et al (2011)^9^	16	26 (6)	7:9	Yes	Intravenous	1·25 mg	Normal saline	PANSS: positive and negative	30 min
Ranganathan et al (2012)^10^	26	27·1 (7·6)	26:4	No	Intravenous	1·89 mg	Vehicle*	PANSS: positive and negative	65 min
Bhattacharyya et al (2009),^11^ Bhattacharyya et al (2012),^42^ Fusar-Poli et al (2009)^43^	15	26·7 (5·7)	15:0	Yes	Oral	10 mg	Flour capsule	PANSS: positive and negative	120 min
Radhakrishnan et al (2015)^44^	23	25·4 (7·4)	21:0	Yes	Intravenous	1·21 mg	Ethanol vehicle*	PANSS: positive, negative, general, and total	70 min
Liem-Moolenaar et al (2010)^18^	11	24·1 (6·7)	11:0	Yes	Inhaled	2 mg, 4 mg, 6 mg	Matching placebo	PANSS: total, positive, and negative	40 min after last dose
Kleinloog et al (2012)^17^	32	22·3 (3·18)	32:0	Yes	Inhaled	2 mg, 4 mg, 6 mg	Placebo THC	PANSS: total, positive, and negative	36 min after last dose
Morgan et al (2018)^7^	48	21·7 (1·8)	34:14	Yes	Inhaled	8 mg	Ethanol vehicle	BPRS: positive and negative	Not recorded
Bossong et al (2009)^13^	7	21·9 (2·7)	7:0	Yes	Inhaled	8 mg	Ethanol vehicle	BPRS: total and positive	21 min

BPRS=Brief Psychiatric Rating Scale. PANSS=Positive and Negative Syndrome Scale. THC=Δ^9^-tetrahydrocannabinol.

* 2 mL 190 proof ethanol vehicle.

† Two independent samples from the same study.

**Figure 2:**
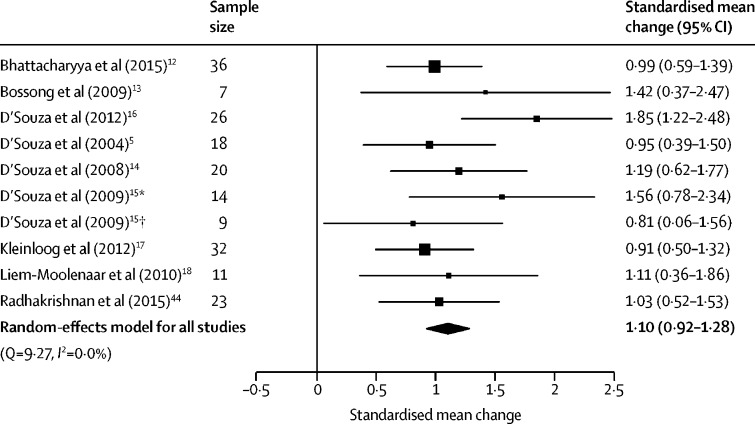
Forest plot of total psychiatric symptom severity following THC relative to placebo The size of the squares reflects the weight attributed to each study. Exact study weights are presented in the appendix (p 13). The diamond denotes the summary effect size for the random-effects model for all studies, and the width of the diamond depicts the overall 95% CI. THC=Δ^9^-tetrahydrocannabinol. *Low cannabis use sample. †High cannabis use sample.

Total symptoms were assessed in nine studies with ten samples (two independent samples included from D'Souza et al^15^), involving 196 participants. THC significantly increased total symptom severity compared with placebo, with a large effect size (SMC 1·10 [95% CI 0·92–1·28], p<0·0001; figure 2). The result remained significant in all iterations of the leave-one-out analysis (SMC ranged from 1·03 [95% CI 0·92–1·36] to 1·15 [0·95–1·35]; appendix p 21).

No between-sample inconsistency was detected (*I*^2^=0%, Cochran's Q=9·27, p=0·41). Egger's test did not identify evidence of publication bias (p=0·14). However, trim-and-fill analysis estimated two missing studies on the left-hand side. The SMC was reduced but remained significant after imputation of the two missing studies (SMC 1·02 [95% CI 0·78–1·25], p<0·0001; appendix p 14).

There were no significant linear relationships between the magnitude of placebo–THC differences and age (n=10, β=0·02 [95% CI −0·07 to 0·11], p=0·68), sex (n=10, β=–0·01 [–0·02 to 0·00], p=0·10), tobacco smoking (n=6, β=–0·02 [–0·06 to 0·02], p=0·30), THC dose (n=6, β=–0·05 [–0·26 to 0·16], p=0·65; including studies of intravenous THC only because of insufficient data for analysis for other routes of administration), or study quality (n=10, β=–0·07 [–0·40 to 0·26], p=0·69). Moreover, the induction of total symptoms was not modified by the use of intravenous or inhaled THC (intravenous *vs* inhaled: Z=–0·90, p=0·37), frequent cannabis use (Z=35, p=0·73), current cannabis use (Z=0·07, p=0·95) or study author (Z=1·06, p=0·29). An insufficient number of studies used BPRS, synthetic THC, or oral THC to enable a moderator analysis of these variables.

**Figure 3:**
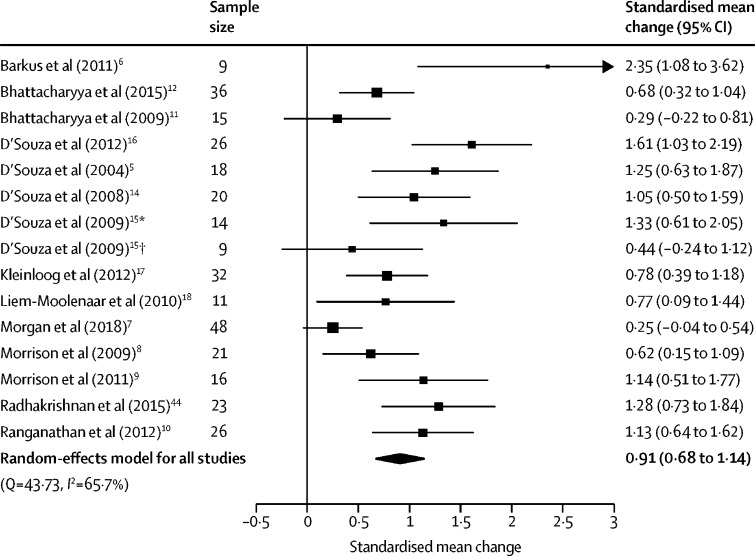
Forest plot of positive symptom severity following THC relative to placebo The size of the squares reflects the weight attributed to each study. Exact study weights are presented in the appendix (p 13). The diamond denotes the summary effect size for the random-effects model for all studies, and the width of the diamond depicts the overall 95% CI. THC=Δ^9^-tetrahydrocannabinol. *Low cannabis use sample. †High cannabis use sample.

Positive symptoms were assessed in 14 studies (15 independent samples) involving 324 participants. THC increased positive symptom severity compared with placebo (SMC 0·91 [95% CI 0·68–1·14], p<0·0001; figure 3). The result remained significant in all iterations of the leave-one-out analysis (SMC ranged from 0·85 [95% CI 0·63–1·07] to 0·96 [0·75–1·18]; appendix p 22).

There was medium between-sample inconsistency (*I*^2^=65·70%, Cochran's Q=43·73, p<0·0001). Egger's test implied significant publication bias (p=0·0007). Trim-and-fill analysis estimated one missing study on the left-hand side (appendix p 14). The SMC was reduced but remained significant after imputation of the missing study (SMC 0·87 [95% CI 0·63–1·11], p<0·0001).

Intravenous THC induced more severe positive symptoms than did inhaled THC (Z=2·34, p=0·014; appendix p 15), and studies completed by the D'Souza group were also associated with more severe positive symptoms than studies by other authors (Z=2·89, p=0·0038; appendix p 15). There was an insufficient number of studies to evaluate the effect of oral THC. There was a negative association between tobacco smoking and positive symptoms induced by THC (n=10, β=–0·01 [95% CI −0·02 to 0·00], p=0·019; appendix p 16). Studies with higher quality were associated with a greater effect on positive symptoms (n=15, β=0·26 [95% CI 0·06–0·47], p=0·011; appendix p 16).

By contrast, there were no significant linear relationships between the magnitude of THC–placebo differences and age (n=15, β=0·09 [95% CI −0·01 to 0·19], p=0·069), sex (n=15, β=–0·01 [–0·02 to 0·01], p=0·27), or dose of THC (n=10, β=–0·01 [–0·21 to 0·18], p=0·91; only reported for studies using intravenous administration). Similarly, frequent cannabis use (Z=0·87, p=0·38), current cannabis use (Z=–1·10, p=0·27), and type of THC (synthetic *vs* purified; Z=–0·73, p=0·47) did not significantly moderate the induction of positive symptoms. An insufficient number of studies used BPRS to enable a moderator analysis of symptom scale used.

**Figure 4:**
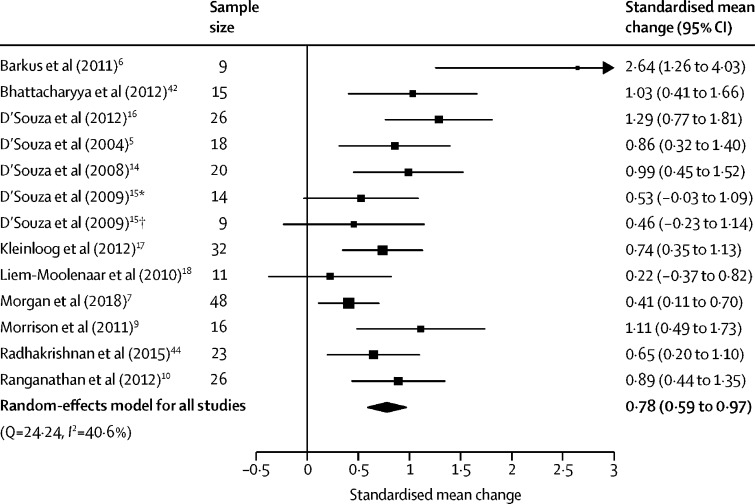
Forest plot of negative symptom severity following THC relative to placebo The size of the squares reflects the weight attributed to each study. Exact study weights are presented in the appendix (p 13). The diamond denotes the summary effect size for the random-effects model for all studies, and the width of the diamond depicts the overall 95% CI. THC=Δ^9^-tetrahydrocannabinol. *Low cannabis use sample. †High cannabis use sample.

Negative symptoms were assessed in 12 studies (13 independent samples) involving 267 participants. THC increased the severity of negative symptoms compared with placebo, with a large effect size (SMC 0·78 [95% CI 0·59–0·97], p<0·0001; figure 4). The result remained significant in all iterations of the leave-one-out analysis (SMC ranged from 0·72 [95% CI 0·55–0·90] to 0·83 [0·66–1·00]; appendix p 22). THC induced a greater effect on positive symptoms than on negative symptoms (Z=2·06, p=0·039), although this finding did not remain significant when refitting the model with a lower between-symptom correlation coefficient (*r*=0·1, Z=1·53, p=0·13; appendix p 20).

There was medium between-sample inconsistency (*I*^2^=40·57%, Cochran's Q=24·24, p=0·019). Egger's test implied significant publication bias (p=0·0069). Trim-and-fill analysis did not identify any missing studies (appendix p 17).

As with positive symptoms, intravenous THC induced greater negative symptoms than did inhaled THC (Z=2·43, p=0·015; appendix p 17). An insufficient number of studies used oral THC to evaluate its modifying effects. Higher mean age of the sample predicted greater negative symptoms induced by THC (n=13, β=0·08 [95% CI 0·01–0·15], p=0·022; appendix p 18).

There were no significant linear relationships between the magnitude of THC–placebo differences and sex (n=13, β=–0·00 [95% CI −0·01 to 0·01], p=0·89), tobacco smoking (n=8, β=–0·00 [–0·01 to 0·01], p=0·41), THC dose (n=9, β=0·03 [–0·12 to 0·18], p=0·73; only assessed in studies of intravenous THC), or study quality (n=13, β=–0·00 [–0·21 to 0·20], p=0·99). Similarly, frequent cannabis use (Z=–0·23, p=0·82), current cannabis use (Z=–0·94, p=0·35), type of THC (synthetic *vs* purified; Z=–1·35, p=0·18), and study author (Z=0·062, p=0·95) did not significantly moderate the induction of negative symptoms. An insufficient number of studies used BPRS to enable a moderator analysis of symptom scale used.

**Figure 5:**
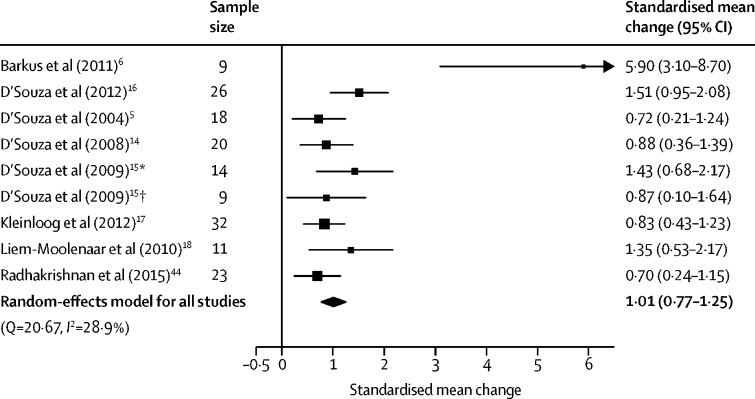
Forest plot of general psychiatric symptom severity following THC relative to placebo The size of the squares reflects the weight attributed to each study. Exact study weights are presented in the appendix (p 13). The diamond denotes the summary effect size for the random-effects model for all studies, and the width of the diamond depicts the overall 95% CI. THC=Δ^9^-tetrahydrocannabinol. *Low cannabis use sample. †High cannabis use sample.

General symptoms were assessed in eight studies (nine independent samples) involving 162 participants. THC significantly increased general symptoms compared with placebo with a large effect size (SMC 1·01 [95% CI 0·77–1·25], p<0·0001; figure 5). The result remained significant in all iterations of the leave-one-out analysis (SMC ranged from 0·90 [95% CI 0·70–1·11] to 1·08 [0·81–1·35]; appendix p 22). No significant differences were found between the effect on general symptoms and positive (Z=0·44, p=0·66) or negative symptoms (Z=1·90, p=0·058), although the latter became significant when refitting the model with a higher between-symptom correlation coefficient (*r*=0·7, Z=2·01, p=0·044; appendix p 20).

There was medium between-sample inconsistency, with an *I*^2^ value of 28·90% (Cochran's Q=20·67, p=0·0081). Egger's test implied significant publication bias (p=0·0002). Trim-and-fill analysis estimated three missing studies on the left side (appendix p 18). The SMC was reduced but remained significant after imputation of the missing study (SMC 0·85 [95% CI 0·53–1·17], p<0·0001).

There were no significant linear relationships between the magnitude of THC–placebo differences in general symptoms and age (n=9, β=–0·00 [95% CI −0·13 to 0·13], p=0·95), sex (n=9, β=–0·00 [–0·02 to 0·01], p=0·72), tobacco smoking (n=6, β=–0·01 [–0·04 to 0·03], p=0·67), THC dose (n=7, β=–0·08 [–0·33 to 0·17], p=0·52; only assessed in studies of intravenous THC), or study quality (n=9, β=–0·02 [–0·48 to 0·45], p=0·95). Similarly, intravenous and inhaled THC (Z=–0·31, p=0·76), frequent cannabis use (Z=–0·068, p=0·95), current cannabis use (Z=–0·84, p=0·38), and study author (Z=1·06, p=0·29) did not significantly moderate the induction of general symptoms. An insufficient number of studies used BPRS, oral THC, or synthetic THC to enable moderator analyses of these variables.

The effect of CBD on psychopathology compared with placebo was evaluated in two within-person studies and one between-person study (figure 1), with one further study that used the CAPE scale identified by our additional searches (appendix p 19). In the systematic review, there were no significant differences between CBD and placebo in any of the subscales reported (appendix p 11).

Similarly, two within-person and two independent group design studies assessed the effects of CBD on the induction of symptoms by THC (figure 1; table 2; appendix p 12). The first study demonstrated a significant reduction in positive symptoms,^25^ albeit in a modest sample. A further study found no significant effect of CBD in the main analysis, but an exploratory analysis demonstrated a significant reduction in symptoms when restricted to participants who had an increase of 3 or more points on the psychotic scale with THC alone.^20^ By contrast, two other studies showed no significant effect of CBD on THC-induced positive, negative, or total symptoms.^7^, ^26^ However, one of these studies did not show a significant increase in positive symptoms when THC was administered alone.^7^

**Table 2:** Studies evaluating the effect of CBD on the psychotomimetic properties of THC in healthy individuals

	**Sample size and study design**	**Mean age (SD), years**	**Male:female**	**Randomised group or order**	**Route**	**Dose**	**Placebo condition**	**Scale and subscales**	**Effect**
Bhattacharyya et al (2010)^25^	6 within person	25·6 (8·2)	3:3	Yes	Intravenous	1·25 mg THC, 5 mg CBD	Vehicle (ethanol)	PANSS: positive	Reduced
Morgan et al (2018)^7^	48 within person	21·7 (1·8)	34:14	Yes	Inhaled	8 mg THC, 16 mg CBD	Ethanol vehicle	BPRS: positive and negative	No change
Englund et al (2013)^20^	48 between person; 22 CBD; 26 placebo	Active CBD: 25 (3); placebo CBD: 26 (4)	Active: 13:9; placebo 14:12	Yes	Intravenous THC, oral CBD	1·5 mg THC, 600 mg CBD	Matching capsules	PANSS: positive	No change or reduced
Mueller et al (2016)^26^	30 (15 in each group) between person	NR	NR	Yes	Oral	20 mg THC, 800 mg CBD	NR	PANSS: positive and total	No change

BPRS=Brief Psychiatric Rating Scale. CBD=cannabidiol. PANSS=Positive and Negative Syndrome Scale. NR=not recorded. THC=Δ^9^-tetrahydrocannabinol.

## Discussion

We demonstrate that acute administration of THC induces significant increases in positive, negative, general, and total symptoms with large effect sizes in adults with no history of psychotic or other major psychiatric disorders. Notably, effect sizes were greater for positive symptoms than for negative symptoms but not for general symptoms, indicating that THC induces positive symptoms to a greater extent than negative symptoms. This result is consistent with findings that symptom severity is greater for positive than negative symptoms in cannabis users.^45^ Our findings extend these previous findings to show that this is also the case in experimental settings. Although the effect of THC on symptoms remained significant for different routes of administration, the effects of intravenous administration were more pronounced than those of inhalation. This finding indicates that the route of administration modifies THC's effects, although this association might be confounded by dose or rate of administration. We were unable to test this formally because of a lack of power. It would be useful for future studies to investigate this. Although positive symptoms were also more pronounced in studies by the D'Souza group, all of these studies used intravenous THC, which, as intravenous administration is associated with larger effects, could underlie this association. In addition, lower rates of tobacco use and higher study quality were associated with greater positive symptoms, whereas higher mean age was associated with greater induction of negative symptoms. Notably, positive symptoms were not moderated by dose or previous cannabis use. This result contrasts with findings from several primary studies that report dose–response relationships and evidence of a blunted psychotomimetic effect among regular cannabis users.^5^, ^14^ The lack of relationship in our analysis might reflect limited power in this analysis and suggests further work is needed to investigate these factors. There was an insufficient number of studies to meta-analyse the effect of CBD alone or the moderating effects of CBD on THC-induced symptoms. Our systematic review found that there is no evidence for CBD having a significant effect on positive, negative, general, or total symptoms. Similarly, although a single, small study (n=6) reported a significant reduction in THC-induced positive symptoms by CBD,^25^ three larger studies failed to replicate this finding.^7^, ^20^, ^26^

A strength of our analysis is that it focused on experimental studies with placebo control conditions, which avoids the risk of reverse causality and residual confounding factors associated with observational studies of psychotic symptoms in cannabis users.^46^ However, a number of study limitations should be considered in evaluating our findings. Many of the meta-regression analyses comprised fewer than ten studies and so were underpowered to detect small-to-moderate effects. Thus, we cannot exclude a modifying effect of some variables on our findings, in particular tobacco use and THC dose on the induction of total, negative, or general symptoms by THC, or age or gender on general symptoms, although our analyses suggest that any potential effects are not large. There was a preponderance of male-dominated samples in the studies. Although no effect of sex was identified, future studies should include more females to ensure generalisability. We identified potential publication bias in positive, negative, and total symptom domains. This bias might be due to selective reporting of symptom scales with significant findings. Nevertheless, effect sizes for positive symptoms were positively associated with study quality, suggesting that our findings might be underestimating effect size as a result of the inclusion of lower quality studies that have smaller effect sizes, and findings remained largely unchanged after adjusting for putatively missing studies. Finally, we used summary symptom measures that combine scores across several symptoms, which precludes the analysis of individual symptoms. Future work should focus on the effect of cannabinoids on specific symptoms of interest, such as hallucinations and delusions.

The studies we analysed used doses of THC ranging from 1·25 mg to 10 mg, leading to peak THC blood concentrations of 4·56–5·1 ng/mL when orally administered^12^, ^25^ and 110–397 ng/mL following intravenous or inhaled administration.^9^, ^16^ These concentrations are similar to those seen shortly after smoking a typical cannabis joint containing 16–34 mg of THC.^5^, ^40^, ^47^ Thus, our findings have implications for the 188 million people who use cannabis and other THC-containing cannabinoids worldwide each year, and for the therapeutic use of cannabis and its derivatives. They indicate that use of THC-containing products could induce a range of psychiatric symptoms, including psychotic symptoms such as hallucinations and paranoia.^5^ In addition to causing distress, these symptoms might lead to harmful behaviours, including self-harm, agitation, and violence.^48^ It has been argued that CBD in cannabis counters the psychotic and other effects of THC,^23^ however, concurrent CBD administration did not significantly reduce the induction of symptoms in three of four studies identified.^7^, ^20^, ^26^ Thus, currently, the experimental evidence base is not strong for increasing CBD content in cannabis to counter the effects of THC.

Our finding that the induction of psychotic symptoms was lower in people with higher tobacco use could suggest that tobacco use is a protective factor, but further work is needed to test causality and this finding should not be taken as a recommendation to use tobacco to counter the effects of THC. Tobacco smoking is associated with lower brain CB1 receptor levels,^49^ which could mean smokers are less sensitive to the effects of THC. The association between lower induction of psychotic symptoms by THC and higher tobacco use might also relate to the upregulation of UDP-glucuronosyltransferase by nicotine,^50^ which in turn is implicated in the metabolism of THC.^51^ Finally, although this study investigated the acute effects of THC, the magnitude and consistency of effects across symptom domains add to the evidence implicating the endocannabinoid system and cannabis use in the pathophysiology of schizophrenia and other psychotic disorders.^52^, ^53^, ^54^

In conclusion, these findings demonstrate that the acute administration of THC induces positive, negative, and general psychiatric symptoms with large effect sizes. By contrast, CBD does not induce psychiatric symptoms, and there is inconclusive evidence that it moderates the induction of psychiatric symptoms by THC. These effects are larger with intravenous administration than with inhaled administration, and tobacco smokers have less severe positive symptoms. These findings highlight the acute risks of cannabis use, which are highly relevant as medical, societal, and political interest in cannabinoids continues to grow.
