# Supplementary material for: Psychiatric symptoms caused by cannabis constituents: a systematic review and meta-analysis
Source: Lancet Psychiatry. 2020 Apr;7(4):344–53. doi: 10.1016/S2215-0366(20)30074-2 (PMC7738353; doi:10.1016/S2215-0366(20)30074-2)
Supplement: Supplementary appendix [file mmc1.pdf]

THE LANCET
Psychiatry

Supplementary appendix

This appendix formed part of the original submission and has been peer reviewed. We post it as supplied by the authors.

Supplement to: Hindley G, Beck K, Borgan F, et al. Psychiatric symptoms caused by cannabis constituents: a systematic review and meta-analysis. *Lancet Psychiatry* 2020; published online March 17. https://doi.org/10.1016/S2215-0366(20)30074-2.

Supplementary Information

Supplementary Methods

Supplementary Table 1 Table summarising timings of peak symptoms and duration of symptoms for studies included in meta-analysis of acute administration of THC
Supplementary Table 2. Raw data used in positive symptom analysis for healthy participants
Supplementary Table 3. Raw data used in negative symptom analysis for healthy participants
Supplementary Table 4. Raw data used in general symptom analysis for healthy participants
Supplementary Table 5. Raw data used in total symptom analysis for healthy participants
Supplementary Table 6. Summary of study characteristics: within person studies examining acute THC administration in healthy controls- study information
Supplementary Table 7. Newcastle-Ottawa Scale for studies included in meta-analysis of acute administration of THC in healthy participants
Supplementary Table 8. Summary of study characteristics: studies examining the effect of CBD alone in healthy controls
Supplementary Table 9. Summary of study characteristics: studies examining the modifying effect of CBD on THC-induced symptoms in healthy controls
Supplementary Table 10 Study weights employed in meta-analysis
Supplementary Table 11: Table summarising study samples and designs involving healthy individuals receiving THC and placebo using CAPE as the outcome measure.

Supplementary Figure 1. Funnel Plot for total symptoms
Supplementary Figure 2. Funnel Plot for positive symptoms
Supplementary Figure 3. Sub-group analysis of effect of route of administration on positive symptoms
Supplementary Figure 4 Sub-group analysis of effect of study author (D'Souza group vs other) on positive symptoms of schizophrenia
Supplementary Figure 5. Meta-regression showing a negative relationship between percentage current smokers and the induction of positive symptoms by THC
Supplementary Figure 6 Meta-regression showing a positive relationship between Newcastle Ottawa Score for study quality and the induction of psychotic symptoms by THC (n=15, β=0.26, p=0.011).
Supplementary Figure 7. Funnel Plot for negative symptoms
Supplementary Figure 8. Sub-group analysis of the moderating effect of route of administration on THC induced negative symptoms
Supplementary Figure 9. Meta-regression showing a positive relationship between mean age and the induction of negative symptoms
Supplementary Figure 10 Funnel Plot for general symptoms
Supplementary Figure 11: Search process summarising the review and exclusion of papers using CAPE/SAPS and SANS

Appendix 1. Re-fitting the model using r_i = 0.1
Appendix 2. Re-fitting the model using r_i = 0.7
Appendix 3. Peak symptoms and duration of symptoms following the acute administration of THC
Appendix 4. Sensitivity analysis excluding potentially duplicated sample
Appendix 5. Re-fitting the model comparing symptom domains using r_i = 0.1-0.7
Appendix 6. Repeating analysis of frequent cannabis use with lower estimate
Appendix 7. Repeating analysis of frequent cannabis use with higher estimate
Appendix 8. Supplementary analysis including studies using the CAPE scale
Appendix 9. Leave one-out analysis full results

Supplementary Methods

1. Search and data extraction

Data were extracted by two authors independently (G.H. and K.B.). Plot digitizer software (https://automeris.io/WebPlotDigitizer/) was used to extract data when only available in plot format.(1) If more than one dose or time-point was reported, the data for the maximum dose or the time-point associated with the highest mean symptom score was extracted since we aimed to determine the maximum possible effect. Variables extracted were author, year of publication, number of completed participants, mean age, proportion male, percentage current tobacco smokers, mean total lifetime cannabis exposures, details of control condition and randomisation procedure, inclusion and exclusion criteria, route and dose of THC, symptom measure used and subscales reported, timing of measure relative to administration of THC and mean and SD of symptom scales. If dose was presented as mg/kg, the mean dose delivered was calculated by multiplying the dose/kg by the mean weight in kg.

If total cannabis exposure was reported as a categorical variable with ranges, means were estimated by multiplying the midpoint of each range by the frequency within each range, summing the answer for each bin and dividing by the total number of participants. Since the largest category was often "$>z$", where z represents a given number of total cannabis exposures, we used $1.5z$ as the equivalent of the midpoint for the main analysis and performed a sensitivity analysis substituting $1.5z$ with z and $2z$ (see Appendices 6 and 7). As details regarding cannabis use were often reported with low precision, we also dichotomised this variable to reflect high and low frequency of cannabis use (frequent ≥ 100 mean total uses, infrequent ≤ 100 mean total uses), in line with previous literature.(2)

Although not included in the initial protocol, an additional search was made for alternative scales measuring similar constructs: the scale for the assessment of positive symptoms (SAPS), scale for the assessment of negative symptoms (SANS) and the community assessment of psychic experience scale (CAPE). The search terms were identical to the PANSS/BPRS search besides the scales themselves: MEDLINE (from 1 January 1946 to 21 May 2019), EMBASE (from 1 January 1974 to 21 May 2019) and PsychINFO (from 1 January 1806 to week 21 May 2019) were searched. The following keywords were used: (THC OR tetrahydrocannabinol OR 9THC OR 9tetrahydrocannabinol OR delta9THC OR d9THC OR delta9tetrahydrocannabinol OR dronabinol OR marinol OR bedrobinol OR anandamide OR methanandamide OR "WIN,55,212-2" OR ACPA OR CP55940 OR bedrocan OR spice OR JWH-018 OR AM251 OR SR161716A OR rimonabant OR cannabidiol OR CBD OR cannabinoid) AND (SAPS OR "scale for the assessment of positive symptoms" OR SANS OR "scale for the assessment of negative symptoms" OR CAPE OR "community assessment of psychic experience"). The findings from this search are summarised in Appendix 8, Supplementary Figure 11 and Supplementary Table 11.

2. Analysis of moderating variables

In order to evaluate potential modifiers of THCs effects on psychopathology, we conducted secondary subgroup analyses of the following variables thought to potentially modulate sensitivity to THC's pharmacodynamic action or the endocannabinoid system, or key methodological factors. In order to investigate the association between the effect size and continuous variables, meta-regression analyses were conducted. We conducted meta-regressions if n of studies >5. Variables included were mean age(3), percentage male gender(4), and percentage tobacco smokers(5) and Newcastle-Ottawa Scale score (study quality). We grouped studies by route of administration in the analysis of dose as there is currently no standardised method to calculate THC dose equivalence for different routes of administration. We conducted subgroup-analyses for categorical variables. We first conducted a random effects meta-analysis for each subgroup. We then compared subgroup estimates in a fixed effects model with a Wald-type test. Variables were selected on the basis of prior evidence that they might influence the effect of THC, and were current use (comparing studies where subjects had confirmed recent use with a positive urine drug screen for cannabis vs studies where subjects had confirmed abstinence from recent cannabis with a negative urine drug screen for cannabis at screening)(6), frequency of use (mean total exposures>100 vs mean total exposures<100)(6), route of administration (oral vs inhaled vs IV)(7), type of THC (purified vs synthetic), symptom scale (BPRS vs PANSS) and study author (D'Souza et al. or group vs other)(8).

3. Comparing effect of THC on positive, negative and general symptoms

To determine whether the effect of THC was greater on positive, negative or general symptoms a multivariate meta analytic approach was adopted using an unstructured variance-covariance matrix. As within study correlations for positive, negative and general symptom scores are not reported we estimated the correlation coefficient to be 0.5 based on prior studies (9). We performed a sensitivity analysis to evaluate the influence of this assumption on these results by re-fitting our model using r values of 0.1 and 0.7 (See Appendix 6).

Table 1: Timepoints measured, timings at peak symptoms and timings at resolution of symptoms for studies included in meta-analysis. Resolution of symptoms determined by qualitative evaluation of graphs comparing THC score to placebo score at corresponding timepoint. Scale is PANSS unless otherwise stated. *Timepoints are taken from a graph. These timepoints do not correspond to timepoints described in the methods and the scale on the graph has varying intervals (i.e. is not linear).

	Route	Symptom domains	Timepoints (mins)	Peak (mins)	Time at resolution of symptoms (mins)	Comment
**Bhattacharyya 2015**	Oral	Positive, total	60, 120, 180	120	Not resolved	
**Bhattacharyya 2012**	Oral	Positive, negative	60, 120	120	Not resolved	
**Bossong 2009**	Inhaled	BPRS Total	21, 102	21	Not resolved	
**Liem-Moolenaar 2010**	Inhaled	Positive, negative, general, total	40 after last dose	40	Not resolved	Multiple THC doses
**Kleinloog 2012**	Inhaled	Positive, negative, general, total	36 after last dose	36	Not resolved	Multiple THC doses
**Morgan 2018**	Inhaled	BPRS Positive, negative	Not recorded	N/a	N/a	
**D'Souza 2012**	IV	Positive, negative, general, total	10, 80	10	Not resolved	
**D'Souza 2004**	IV	Positive, negative, general, total	10, 80, 200	10 (positive, general, total)<br>80 (negative)	200	
**D'Souza 2008**	IV	Positive, negative, general, total	10, 80, 200	10	200	
**D'Souza 2009a**	IV	Positive, negative, general, total	15, 65	15	Not resolved	
**D'Souza 2009b**	IV	Positive, negative, general, total	15, 65	15	Not resolved	
**Radhakrishnan 2015**	IV	Positive, negative, general, total	70, 240	70	240	
**Barkus 2011**	IV	Positive, negative, general	30, 80, 120	30	240 (positive)<br>80 (negative and general)	
**Morrison 2009**	IV	Positive, CAPE negative	30, 80, 120	30	120	
**Morrison 2011**	IV	Positive, negative	30, 90	30	90 (Negative)<br>Positive not resolved by 90	
**Ranganathan 2012**	IV	Positive, negative	10, 65, 120, 250	120 (positive)<br>65 (negative)	250	Timepoints unclear*

Supplementary Table 2. Raw data used in positive BPRS and PANSS analysis for healthy participants

author	year	time	n1i	m1i	sd1i	n2i	m2i	sd2i
Barkus	2011	30	9	13.37	2.4	9	7.12	0
Bhattacharyya	2015	120	36	9.6	3.62367	36	7.26	0.582702
D'Souza	2012	10	26	12.65	3.19	26	7.96	2.18
D'Souza	2004	10	18	9.966837	2.71207595	18	6.813776	0.82992611
D'Souza	2008	10	20	10.52	2.61	20	8.05	1.5
D'Souza	2009a	15	14	9.48755251	1.51	14	7.6335764	0.73450605
D'Souza	2009b	15	9	8.54065211	1.27455069	9	7.991526	0.866052
Morrison	2009	30	21	10.41396	5.28399034	21	7.0005403	0
Morrison	2011	30	16	11.3133161	3.5703548	16	7.036427	0
Ranganathan	2012	120	26	9.68	1.93	26	7.726444	1.03775281
Bhattacharyya	2009	120	15	9.58847489	8.27122099	15	7.05801	0.24986901
Morgan	2018	NR	48	6.6870748	1.03687393	48	6.42857	1
Liem-Moolenaar	2010	40	11	10.63	3.96	11	7.72	1.27
Kleinloog	2012	36	32	9.07	2.29	32	7.47	0.9
Radhakrishnan	2015	70	23	10.87	2.44	23	8.04	1.46

author	year	route	thc_drob	dose	prev_cannabis_mean	frequent_use	current_use	tobacco_current	age_m	Male	panss_bprs
Barkus	2011	1	0	2.5	153	1	0		26.3	100%	1
Bhattacharyya	2015	0	1	10	10.8333	0	0	25%	25.97	100%	1
D'Souza	2012	1	1	2.133	318	1		15.79%	25.92	65.3846154%	1
D'Souza	2004	1	1	5	60.77	0	0	23.81%	29	63.6363636%	1
D'Souza	2008	1	1	5	147.51	1	1		24.8	70%	1
D'Souza	2009a	1	1	2.0774	48.86	0	0	0%	25.85	78.5714286%	1
D'Souza	2009b	1	1	2.1125	141.72	1	1	11.11111111%	22.66	100%	1
Morrison	2009	1	0	2.5			0		28	100%	1
Morrison	2011	1	0	1.25	40	0	0		26	43.75%	1
Ranganathan	2012	1	1	1.89				26.66666667%	27.14	86.6666667%	1
Bhattacharyya	2009	0	1	10	8	0	0	46.66666667%	26.7	100%	1
Morgan	2018	2	1	8			1	70.83333333%	21.705	70.83%	0
Liem-Moolenaar	2010	2	1				0		24.1	100%	1
Kleinloog	2012	2	1		292.2	1	0	0%	22.3	100%	1
Radhakrishnan	2015	1	1	1.2147	296.75	1		14.8148148%	26.26	100%	1

Supplementary Table 3. Raw data used in negative BPRS and PANSS analysis for healthy participants

author	year	time	n1i	m1i	sd1i	n2i	m2i	sd2i	route	thc_drob	Dose
Barkus	2011	30	9	8.57	0.54	9	6.99	0	1	0	2.5
D'Souza	2012	10	26	11.73	3.56	26	7.58	2.3	1	1	2.133
D'Souza	2004	80	18	10.54235	6.31486836	18	5.29539162	1.06846315	1	1	5
D'Souza	2008	10	20	8.44	2.14	20	6.5	0.74	1	1	5
D'Souza	2009a	15	14	8.43	1.99	14	7.29	2.08	1	1	2.0774
D'Souza	2009b	15	9	7.78	1.23	9	7.22	0.91	1	1	2.1125
Morrison	2011	30	16	9.980617	2.55898846	16	6.986849	0	1	0	1.25
Ranganathan	2012	65	26	7.28	1.2	26	6.306244	0.80076204	1	1	1.88988636
Bhattacharyya	2012	120	15	9.3956442	2.17196325	15	7.272232	0.59043631	0	1	10
Morgan	2018		48	4.6870748	1.69670242	48	4.047619	1.31965776	2	1	8
Liem-Moolenaar	2010	40	11	11.63	2.91	11	10.45	5.59	2	1	
Kleinloog	2012	36	32	9.89	3.18	32	7.79	1.24	2	1	
Radhakrishnan	2015	70	23	10.87	2.44	23	8.04	1.46	1	1	1.2147

author	year	prev_cannabis_mean	frequent_use	current_use	tobacco_current	age_m	male	panss_bprs
Barkus	2011	153	1	0		26.3	100%	1
D'Souza	2012	318	1		15.79%	25.92	65.3846154%	1
D'Souza	2004	60.77	0	0	23.81%	29	63.6363636%	1
D'Souza	2008	147.51	1	1		24.8	70%	1
D'Souza	2009a	48.86	0	0	0%	25.85	78.5714286%	1
D'Souza	2009b	141.72	1	1	11.1111111%	22.66	100%	1
Morrison	2011	40	0	0		26	43.75%	1
Ranganathan	2012				26.6666667%	27.14	86.6666667%	1
Bhattacharyya	2012	8	0	0	46.6666667%	26.7	100%	1
Morgan	2018			1	70.8333333%	21.705	70.833333%	0
Liem-Moolenaar	2010			0		24.1	100%	1
Kleinloog	2012	292.2	1	0		22.3	100%	1
Radhakrishnan	2015	296.75	1		14.8148148%	26.26	100%	1

Supplementary Table 4 Raw data used in general PANSS analysis for healthy participants

author	year	time	n1i	m1i	sd1i	n2i	m2i	sd2i	route	thc_drob	dose
Barkus	2011	30	9	23.87	0.9	9	16.03	0	1	0	2.5
D'Souza	2012	10	26	25.65	6.3	26	17.12	2.92	1	1	2.133
D'Souza	2004	10	18			18			1	1	5
D'Souza	2008	10	20	19.96	4.44	20	16.45	2.42	1	1	5
D'Souza	2009a	15	14			14			1	1	2.0774
D'Souza	2009b	15	9			9			1	1	2.1125
Liem-Moolenaar	2010	40	11	24.82	4.51	11	19	3	2	1	
Kleinloog	2012	36	32	19.56	3.45	32	17.02	1.73	2	1	
Radhakrishnan	2015	70	23	20.91	4.1	23	18	3.97	1	1	1.2147

author	year	prev_cannabis_mean	frequent_use	current_use	tobacco_current	age_m	male	panss_bprs
Barkus	2011	153	1	0		26.3	100%	1
D'Souza	2012	318	1		15.79%	25.92	65.3846154%	1
D'Souza	2004	60.77	0	0	23.81%	29	63.6363636%	1
D'Souza	2008	147.51	1	1		24.8	70%	1
D'Souza	2009a	48.86	0	0	0%	25.85	78.5714286%	1
D'Souza	2009b	141.72	1	1	11.1111111%	22.66	100%	1
Liem-Moolenaar	2010			0		24.1	100%	1
Kleinloog	2012	292.2	1	0	0%	22.3	100%	1
Radhakrishnan	2015	296.75	1		14.8148148%	26.26	100%	1

Supplementary Table 5. Raw data used in total BPRS and PANSS analysis for healthy participants

author	year	time	n1i	m1i	sd1i	n2i	m2i	sd2i	route
Bhattacharyya	2015	120	36	40.17	9.53892	36	31.67	3.17964	0
D'Souza	2012	10	26	50.04	10.52	26	32.65	5.38	1
D'Souza	2004	10	18	41.94	12.71	18	30.28	2.16	1
D'Souza	2008	10	20	38.92	7.35	20	31	3.48	1
D'Souza	2009a	15	14	36.29	3.92	14	30.64	2.32	1
D'Souza	2009b	15	9	33.44	2.71	9	31.11	2.47	1
Radhakrishnan	2015	70	23	40.83	7.69	23	33.26	6.33	1
Bossong	2009	21	7	23.43	3.41	7	18.14	0.38	2
Liem-Moolenaar	2010	40	11	47.09	8.99	11	36.91	7.85	2
Kleinloog	2012	36	32	38.52	7.7	32	32.28	3.1	2

author	year	thc_drob	dose	prev_cannabis_m	freq_use	current_use	tobacco_current	age_m	male	panss_bprs
Bhattacharyya	2015	1	10	10.83	0	0	25%	25.97	100%	1
D'Souza	2012	1	2.133	318	1		15.79%	25.92	65.3846154%	1
D'Souza	2004	1	5	60.77	0	0	23.81%	29	63.6363636%	1
D'Souza	2008	1	5	147.51	1	1		24.8	70%	1
D'Souza	2009a	1	2.0774	48.86	0	0	0%	25.85	78.5714286%	1
D'Souza	2009b	1	2.1125	141.72	1	1	11.1111111%	22.66	100%	1
Radhakrishnan	2015	1	1.2147	296.75	1		14.8148148%	25.44	100%	1
Bossong	2009	1	8			0		21.9	100%	0
Liem-Moolenaar	2010	1				0		24.1	100%	1
Kleinloog	2012	1		292.2		0		22.3	100%	1

Supplementary Table 6. Summary of Study Characteristics: within person studies examining acute administration of THC in healthy controls

Exclusion criteria:
1 = Past or present psychiatric history (excluding cannabis use disorder)
2 = Recent history of substance abuse (excluding cannabis and nicotine)
3 = Family history of psychosis
4 = Major medical or neurological disorder
5 = Concurrent psychotropic medication use

Study	Blinded	Randomised	THC method of administration/dose	Placebo condition	Symptoms (BPRS/PANSS)	Exclusion Criteria
**Barkus 2011**	Double	Yes	IV bolus<br>2.5mg dronabinol<br><br>Timing: >24 hours<br><br>Fasting: not known	2.5% ethanol + saline	PANSS:<br>Positive<br>Negative<br>General<br><br>Timing: 30 mins after bolus	1,3,4,5
**Bhattacharyya 2015**	Double	Yes	Oral capsule<br>10mg purified THC<br><br>Timing: 1 month<br><br>Fasting: Overnight fasting, light standardised breakfast	Matched placebo capsule	PANSS:<br>Total<br>Positive<br><br>Timing: 120 minutes after administration	1,2,3
**D'Souza 2012**	Double	Yes	IV bolus<br>0.03mg/kg purified THC<br><br>Timing: Not given<br><br>Fasting: Overnight fasting, light standardised breakfast	Ethanol	PANSS: Total, positive, negative<br><br>Timing: 10 mins after bolus	1,2,3,5
**D'Souza 2004**	Double	Yes	IV over 2minutes<br>5mg purified THC<br><br>Timing: >1 week<br><br>Fasting: Overnight fasting + standardised breakfast	Ethanol	PANSS: positive and negative<br><br>Timing 10 mins after bolus for positive, 80 mins for negative	1,2,3
**D'Souza 2008**	Double	Yes	IV over 20 minutes<br>0.0286mg/kg purified THC<br><br>Timing: 125 mins<br><br>Fasting: Overnight fasting + standardised breakfast	Ethanol	PANSS: Total Positive<br><br>Timing: 15 mins after bolus	1,2,3,5
**D'Souza 2009**	Double	No	IV over 20 minutes<br>0.0286mg/kg purified THC<br><br>Timing: 125 minutes<br><br>Fasting: Overnight fasting + standardised breakfast	Matched vehicle	PANSS: positive<br><br>Timing: 15 mins bolus	1,2,3,5
**Morrison 2009/2011**	Double	Yes	IV over 5 minutes<br>2.5mg dronabinol<br><br>Timing: >2 weeks<br><br>Fasting: Not known	Normal saline	Positive (1)<br>Negative<br><br>Timing: 30mins after bolus	1,2,3,4
**Morrison 2011**	Double	Yes	IV over 5 minutes<br>1.25 mg synthetic THC<br><br>Timing: >2 weeks<br><br>Fasting: Not known	Normal saline	PANSS: positive and negative<br><br>Timing: 30 mins after bolus	1,2,3,4

**Ranganathan 2012**	Double	No	IV over 20 minutes<br>0.025mg/kg<br><br>Timing: 120 minutes<br><br>Fasting: Standard light breakfast	Vehicle	PANSS:<br>Positive and negative<br><br>Timing: 65 mins after bolus	1,2,3,5
**Bhattacharyya 2009&2012/Fusar Poli 2009**	Double	Yes	Oral<br>10mg purified THC<br><br>Timing: Not known<br><br>Fasting: Light standardised breakfast	Flour capsule	PANSS:<br>Positive and negative<br><br>Timing: 120 mins after capsule	2
**Rhadakrishnan 2015**	Double	Yes	IV over 10 minutes<br>0.015mg/kg purified THC<br><br>Timing: 1 day<br><br>Fasting: Overnight fasting + light standardised breakfast	Placebo THC	PANSS:<br>Positive, negative, general and total<br><br>Timing: 70 mins after bolus	1,2,3,5
**Liem-Moolenaar 2010**	Double	Yes	Inhaled using volcano vaporizer<br>Consecutive doses of 2mg, 4mg, 6mg purified THC<br><br>Timing: 2 weeks<br><br>Fasting: Light standardised breakfast	Matching placebo	PANSS: Total<br>Positive (1)<br>Negative<br><br>Timing: 40 mins after last dose administered	5
**Kleinloog 2012**	Double	Yes	Inhaled using Volcano vaporizer<br>Consecutive doses of 2mg, 4mg and 6mg purified THC<br><br>Timing: 2 weeks<br><br>Fasting: Not known	Placebo THC	PANSS: total, positive and negative<br><br>Timing: 36 minutes after last dose administered	1,2,3,5
**Bossong 2009**	Double	Yes	Inhaled using Volcano vaporizer<br>8mg purified THC<br><br>Timing: 2 weeks<br><br>Fasting: 4 hours fasting + standardised meal	Ethanol vehicle	BPRS: Total<br><br>Timing: 21 minutes after inhalation	1,3,2,4,5
**Morgan 2018**	Double	Yes	Inhaled using Volcano vaporizer<br>8mg THC<br><br>Timing: 1 week<br><br>Fasting: Not known	Ethanol vehicle	BPRS: Positive and negative<br><br>Scales items not defined.<br><br>Timing not provided	2,4

Supplementary Table 7. Newcastle-Ottawa quality assessment scale for cohort studies

Study	1	2	3	4	5	6	7	8
Barkus 2011	Male university staff	*	*	*	**	*	*	*
Bhattacharyya 2015	Male	*	*	*	**	*	*	Not described
D'Souza 2012	18-35 years old	*	*	*	**	*	*	Lacking detail of reasons for drop-outs
D'Souza 2004	*	*	*	*	**	*	*	Lacking detail of reasons for drop-outs
D'Souza 2008	High cannabis use	*	*	*	**	*	*	Lacking detail of reasons for drop-outs
D'Souza 2009a (Low cannabis use)	*	*	*	*	**	*	*	*
D'Souza 2009b (High cannabis use)	High cannabis use	*	*	*	**	*	*	*
Morrison 2009/2011	Male university staff and students	*	*	*	**	*	*	*
Morrison 2011	University staff and students	*	*	*	**	*	*	*
Ranganathan 2012	*	*	*	*	**	*	*	*
Bhattacharyya 2009/12	Male	*	*	*	**	*	Peak measurement at last timepoint	Not described
Rhadakrishnan 2015	Male	*	*	*	**	*	*	*
Liem-Moolenaar 2010	Male	*	*	*	**	*	*	*
Kleinloog 2012	Male	*	*	*	**	*	*	*
Morgan 2018	Cannabis users, high and low schizotypy	*	*	*	**	*	Single time point	Not described
Bossong 2009	Male	*	*	*	**	*	*	*

1) **Representativeness of the exposed cohort:** * = "truly representative of average healthy individual in the community" or "somewhat representative of the average healthy individual in the community"
2) **Selection of the non-exposed cohort:** * = "Drawn from the same community as the exposed cohort" (within person design)
3) **Ascertainment of exposure:** * = "Secure record" or "Structured interview" (exposure provided as part of experiment)
4) **Demonstration that outcome of interest was not present at start of study**: * = "yes" i.e. SCID used to screen out people with significant psychopathology
5) **Comparability of cohorts on the basis of the design or analysis:**
   a. * = study controls for prior cannabis use
   b. * = study controls for any additional factor/factors
6) **Assessment of outcome**: * = "independent blind assessment" or "record linkage"
7) **Was follow-up long enough for outcomes to occur**: * = "yes" i.e. there were sufficient timepoints to show peak psychopathy
8) **Adequacy of follow-up cohorts**: * = "Complete follow-up – all subjects accounted for" or "subjects lost to follow-up unlikely to introduce bias – number lost - <20% or description provided of those lost"

Table 8: Table summarising studies evaluating CBD's effect on psychopathology compared to placebo

Study	Sample size, n	Age. Mean (S.D)	M:F	Blind	Randomised group/ order	Route	Dose (mg)	Placebo condition	Scale	Effect
Bhattacharyya 2009&2012, Fusar-Poli 2009	15	26.7 (5.7)	15:0	Double	Yes	Oral	600	Flour capsule	PANSS Total positive negative and general	→
Mueller 2016 (Conference abstract)	30 15 in each group Between person	NR	NR	Double	Yes	Oral	800	NR	PANSS Total and positive	→
Hundal 2017	32 Between person 16 in each group	25 (8) placebo 26 (9) CBD	8:8 both placebo and CBD	Double	Yes	Oral	600	Matched placebo	CAPE Total and positive	→
Morgan 2018	48 Within person	21.7 (1.8)	34:14	Double	Yes	Inhaled	16	Ethanol vehicle	BPRS Positive and negative	→

Supplementary Table 9: Summary of study characteristics – studies examining acute administration of THC and CBD in healthy controls

Exclusion criteria:
1 = Past or present psychiatric history (excluding cannabis use disorder)
2 = Recent history of substance abuse (excluding cannabis and nicotine)
3 = Family history of psychosis
4 = Major medical or neurological disorder
5 = Concurrent psychotropic medication use

Study	Design	Blinded	Randomised group/order	THC method of administration/ dose/timing	Placebo condition	Symptoms (BPRS/PANSS)	Exclusion Criteria
**Bhattacharyya 2010**	Within person	Double	Yes	IV over 5 minutes<br>1.25mg THC<br>5mg CBD<br><br>Timing: 2 weeks<br><br>Fasting: Not known	Ethanol vehicle	PANSS: positive<br><br>Timing: 30 and 60 minutes	Not provided
**Morgan 2018**	Within person	Double	Yes	Inhaled using Volcano vaporizer<br>8mg THC<br>16mg CBD<br><br>Timing: 1 week<br><br>Fasting: not known	Ethanol vehicle	BPRS: Positive and negative<br><br>Scales items not defined.<br><br>Timing not known	2,4
**Englund 2013**	Between person	Double	Yes	IV over 10 minutes<br>1.5mg synthetic THC<br>600mg CBD	Matching capsules (CBD) & Ethanol (THC)	PANSS: Positive<br><br>Timing: 10 mins after bolus	1,3,4
**Mueller 2016 (Conference abstract)**	Between person	Double	Yes	Oral<br>20mg THC<br>800mg CBD<br><br>Timing: NR<br><br>Fasting: NR	NR	PANSS: positive and total symptoms<br><br>Timing: not known	NR

Supplementary Table 10: Study weights in meta-analysis (%)

Study	Total symptoms	Positive symptoms	Negative symptoms	General symptoms
Bhattacharyya 2015	20.32	8.65	N/a	N/a
D'Souza 2012	8.05	6.49	7.97	12.53
D'Souza 2004	10.45	6.18	7.60	13.98
D'Souza 2008	9.81	6.84	7.69	14.11
D'Souza 2009a	5.32	5.34	7.28	8.28
D'Souza 2009b	5.70	5.62	5.49	7.87
Radhakrishnan 2015	12.64	6.77	9.40	16.45
Bossong 2009	2.93	N/a	N/a	N/a
Liem-Moolenaar 2010	5.73	5.72	6.66	7.11
Kleinloog 2012	19.05	8.31	10.82	18.95
Barkus 2011	N/a	2.57	1.70	0.72
Morrison 2009	N/a	7.59	N/a	N/a
Morrison 2011	N/a	6.08	6.30	N/a
Ranganathan 2012	N/a	7.34	9.29	N/a
Morgan 2018	N/a	9.38	13.54	N/a
Bhattacharyya 2012	N/a	7.11	6.25	N/a

Supplementary Table 11: Table summarising study samples and designs involving healthy individuals receiving THC and placebo using CAPE as the outcome measure.

Study	Sample size, n	Age. Mean (S.D)	M:F	Blind	Randomised group/ order	Route	Dose (mg)	Placebo condition	Scale	Effect
Morrison 2009	21 Within person	28 (6)	21:0	Double	Yes	IV	2.5	Normal saline	PANSS: Positive	↑
Morrison 2011	19 Within person	NR	19:0	Double	Yes	IV	2.5	2.5% ethanol	CAPE negative	↑
Freeman 2014	82 Between person 41 in each group	30.3 (9.6) placebo, 30.8 (8.5) THC	30:11 (placebo) 26:15 (THC)	Double	Yes	IV	1.5	Normal saline	CAPE Total	↑
Tunbridge 2015	78 Between person 39 in each group	NR (samples split according to COMT genotype)	29:10 (Placebo) 25:14 (THC)	Double	Yes	IV	1.5	Saline	CAPE positive	↑

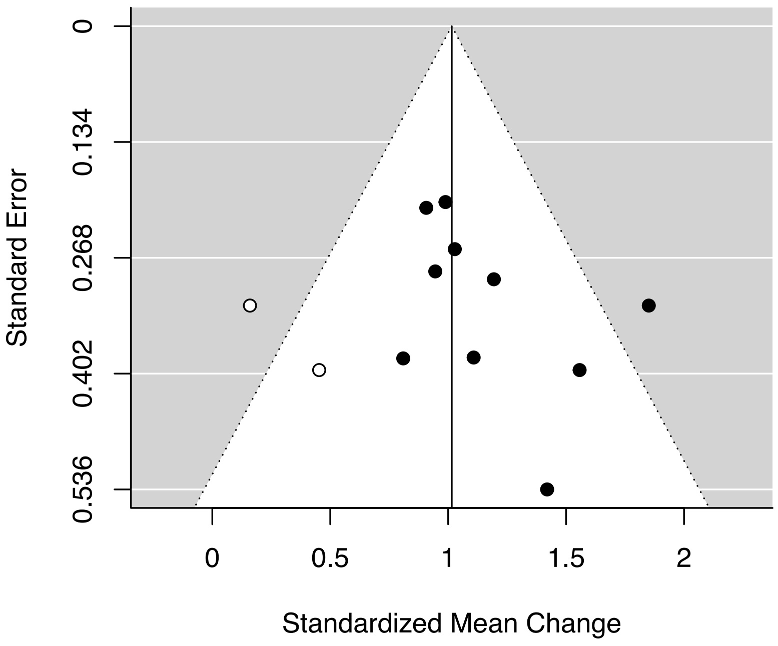

Supplementary Figure 1. Funnel Plot for total symptoms with 2 imputed missing studies

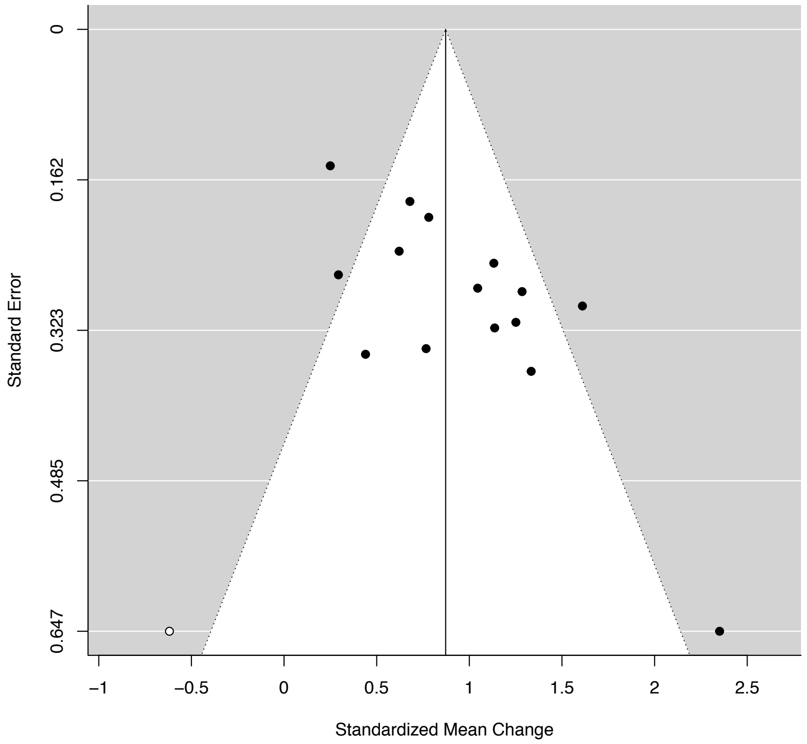

Supplementary Figure 2. Funnel Plot for positive symptoms with a single imputed missing study on the left hand-side

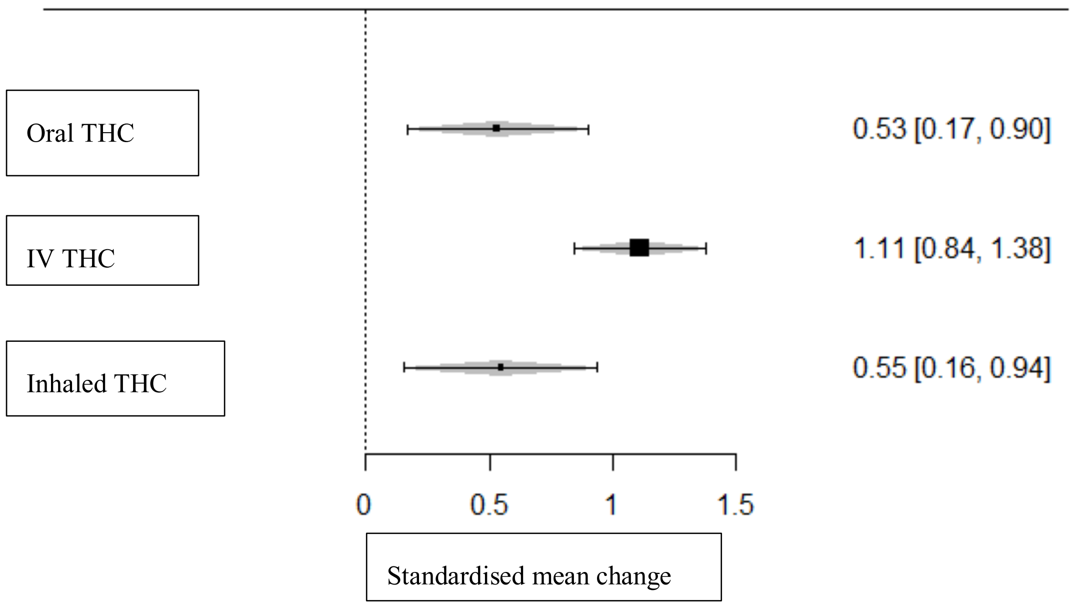

Supplementary Figure 3. Sub-group analysis of effect of route of administration on positive symptoms of schizophrenia.

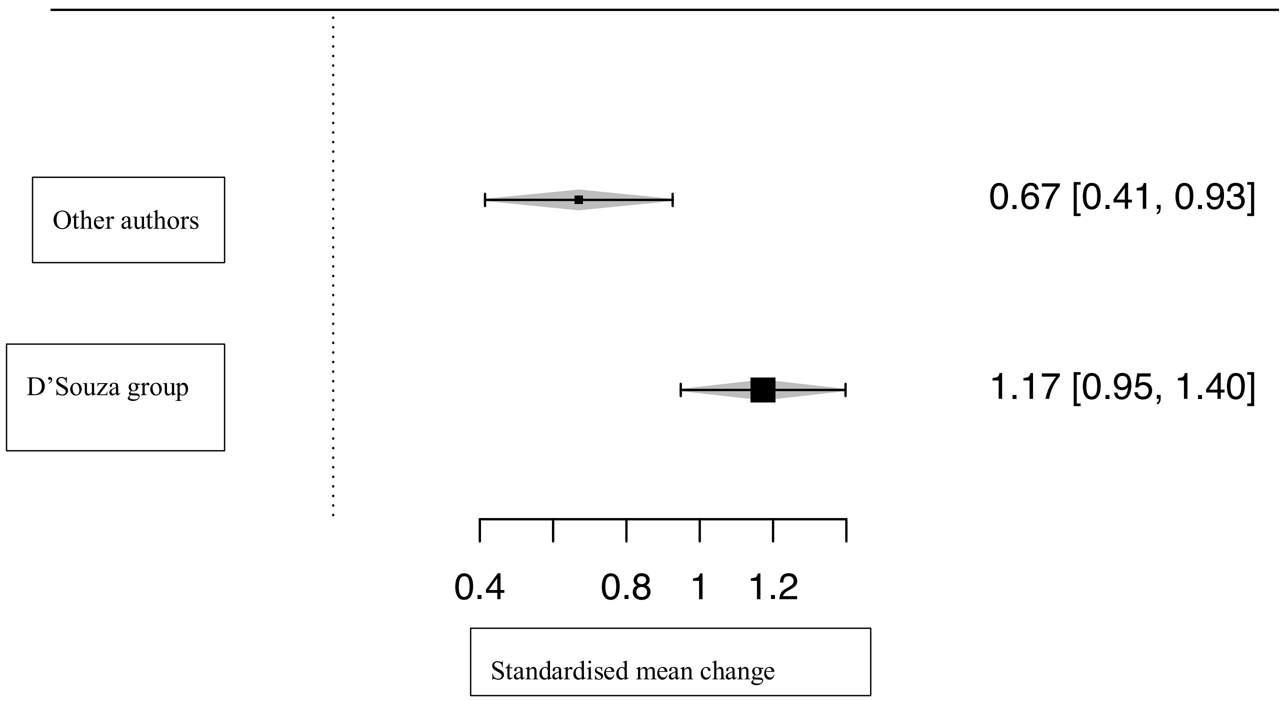

Supplementary Figure 4. Sub-group analysis of effect of study author (D'Souza group vs other) on positive symptoms of schizophrenia.

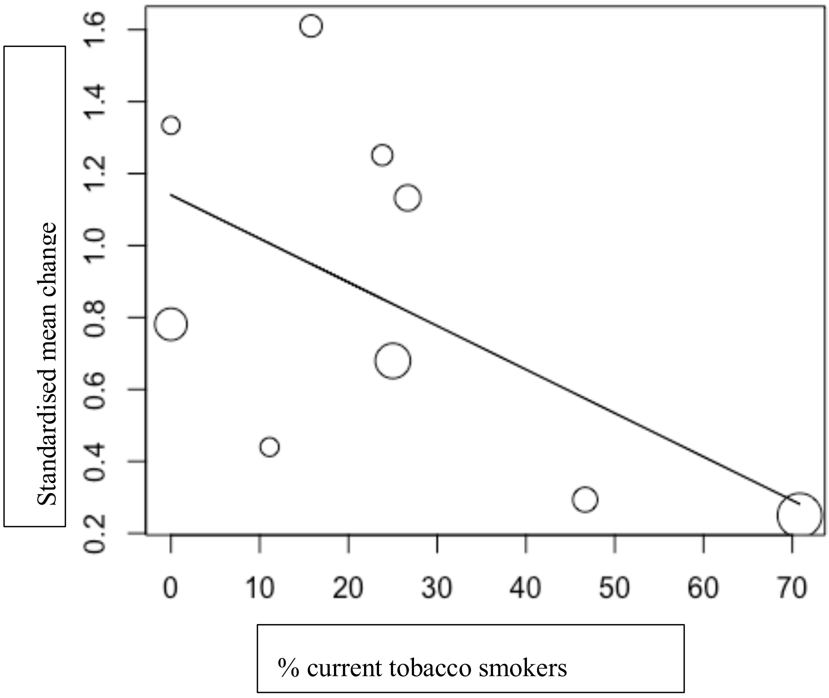

Supplementary Figure 5. Meta-regression showing a negative relationship between percentage current smokers and the induction of positive symptoms by THC (n=10, β=-0.013, p=0.019).

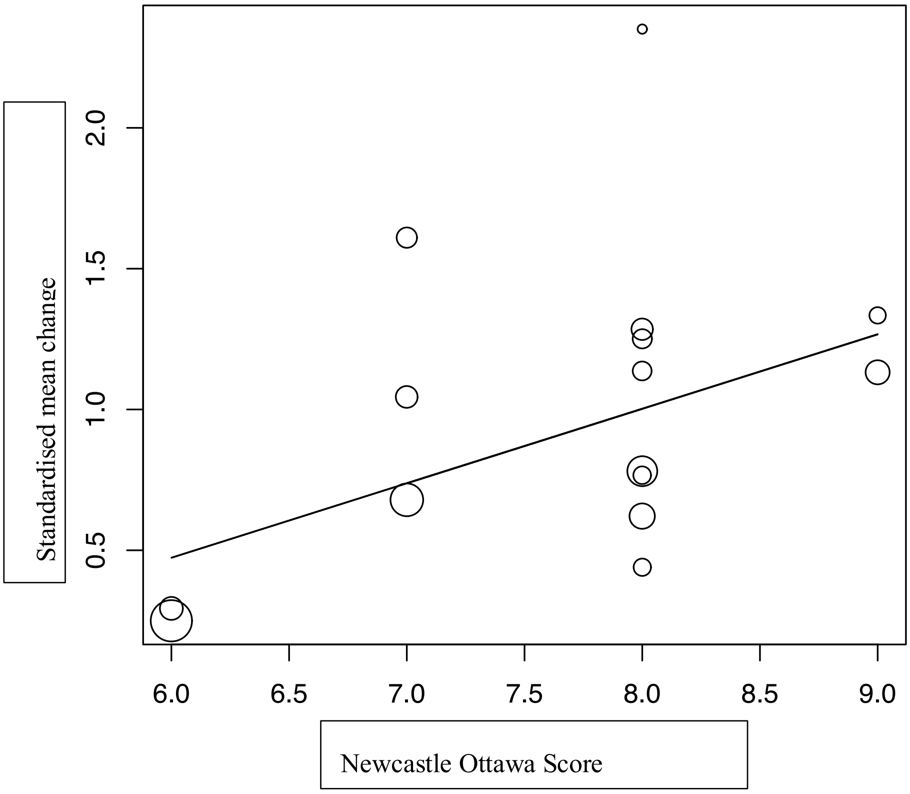

\

Supplementary Figure 6. Meta-regression showing a positive relationship between Newcastle Ottawa Score for study quality and the induction of psychotic symptoms by THC (n=15, β=0.26, p=0.011).

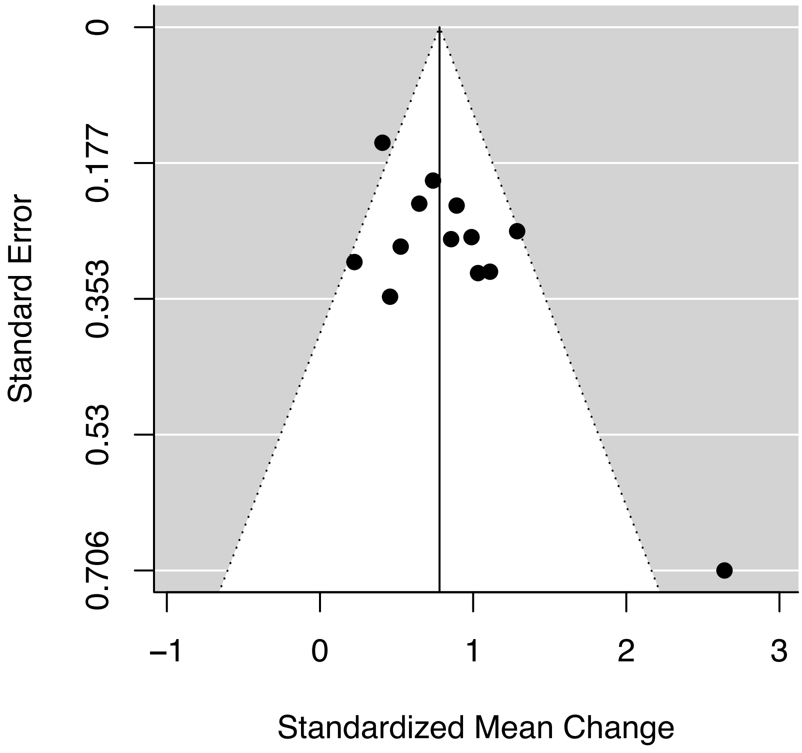

Supplementary Figure 7. Funnel Plot for negative symptoms

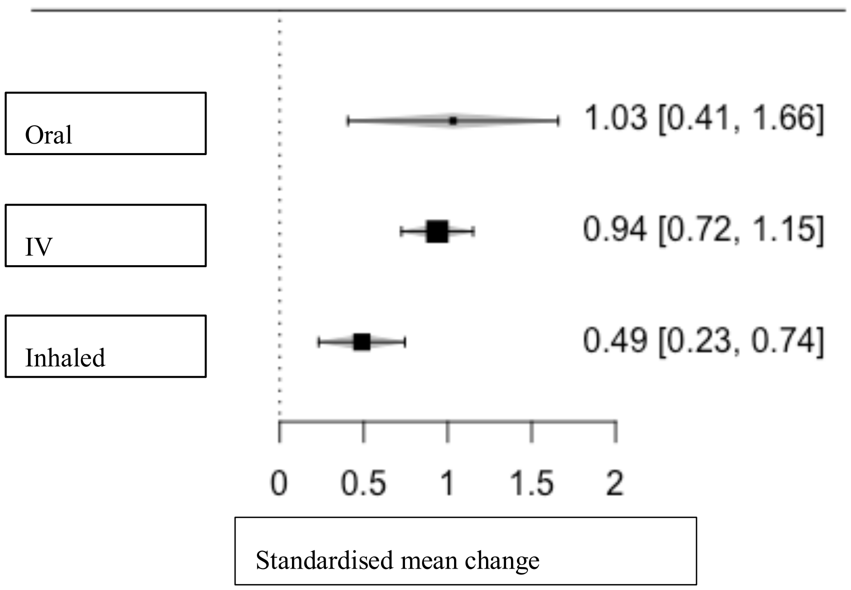

Supplementary Figure 8. Sub-group analysis of the moderating effect of route of administration on THC induced negative symptoms of schizophrenia.

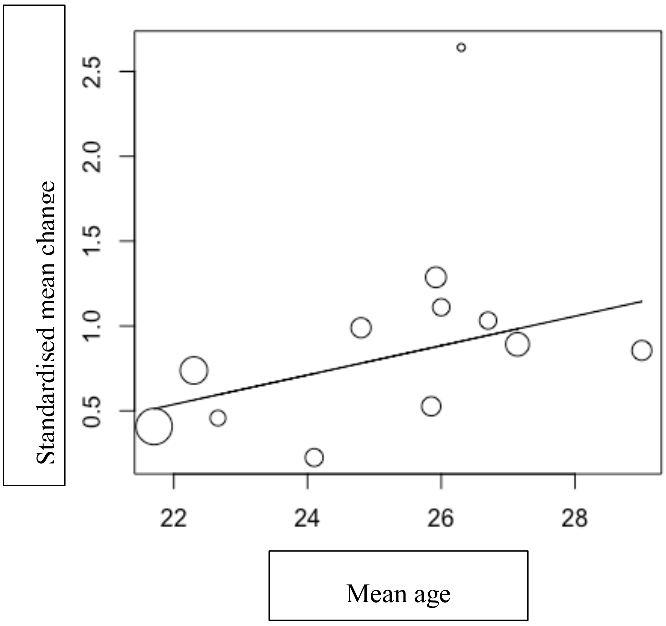

Supplementary Figure 9 Meta-regression showing a positive relationship between mean age and the induction of negative symptoms by THC (n=12, β=0.08, p=0.018).

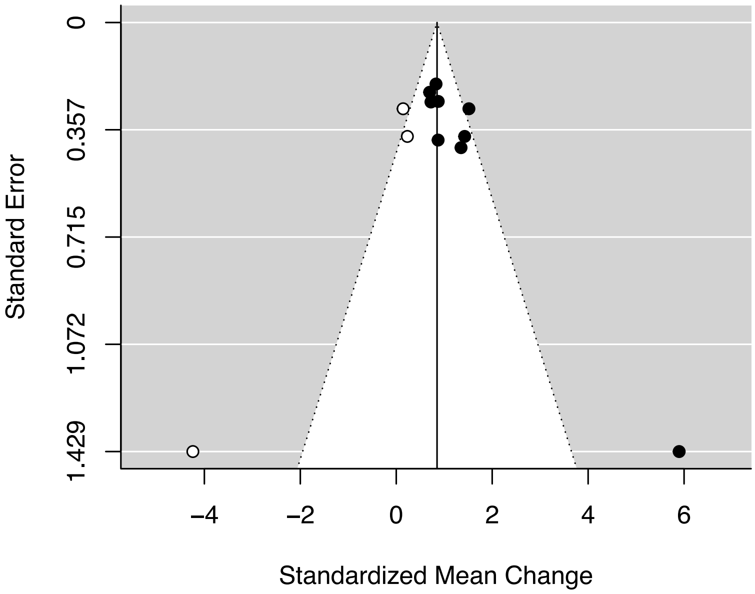

Supplementary Figure 10 Funnel Plot for general symptoms with 3 imputed missing studies

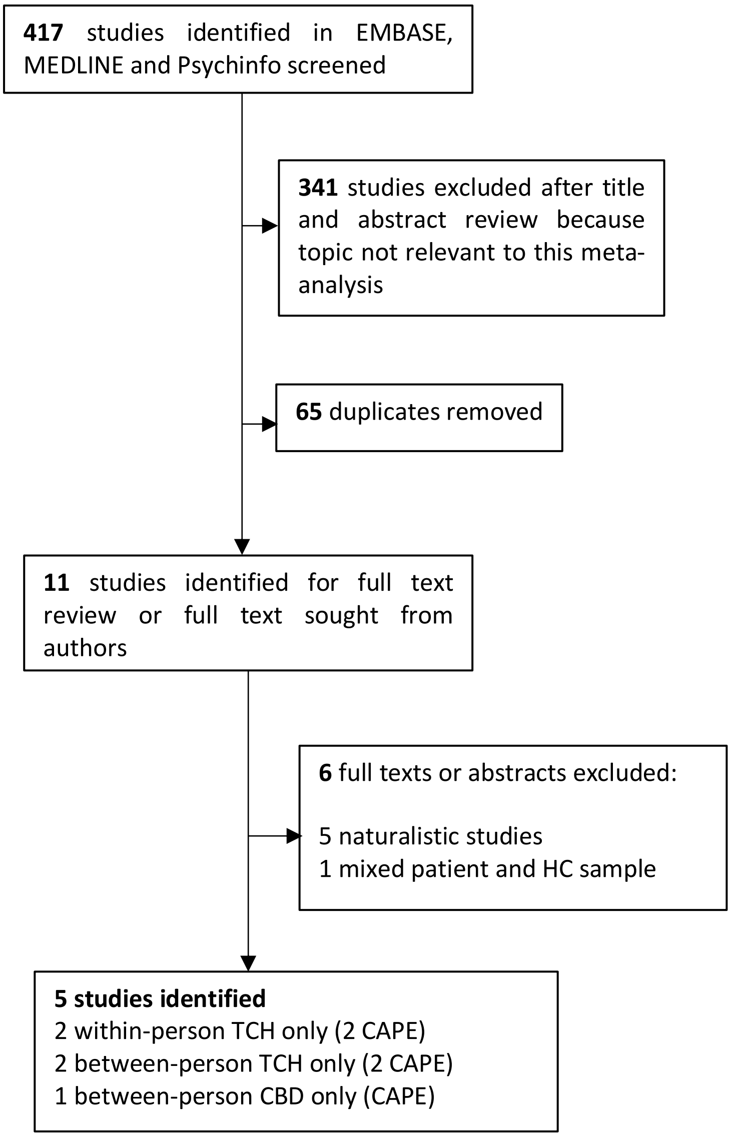

Supplementary Figure 11: Search process summarising the review and exclusion of papers for the CAPE/SANS/SAPS search.

Appendix 1. Re-fitting the model with $r = 0.1$

Acute administration of THC generated an increase in psychopathology in healthy participants, seen as a statistically significant increase in the positive symptoms (Standardised mean change score = 0.77, 95% CI, 0.57 to 0.96, P= <0.0001), negative symptoms (Standardised mean change score = 0.65, 95% CI, 0.48 to 0.83, p< 0.0001), general symptoms (Standardised mean change score = 0.81, 95% CI, 0.61 to 1.02, p< 0.0001) and total symptoms (Standardised mean change score =0.92, 95% CI, 0.75 to 1.09, P<0.0001) compared with a placebo condition.

Appendix 2. Re-fitting the model with $r = 0.7$

Acute administration of THC generated an increase in psychopathology in healthy participants, seen as a statistically significant increase in the positive symptoms (SMC = 1.03, 95% CI, 0.76 to 1.31 , P= <0.0001), negative symptoms (SMC= 0.89, 95% CI, 0.69 to 1.10, p< 0.0001), general symptoms (SMC = 1.17, 95% CI, 0.91 to 1.43, p< 0.0001) and total symptoms (SMC=1.29, 95% CI, 1.06 to 1.52, P<0.0001) compared with a placebo condition.

Appendix 3. Summary of timings of peak symptoms and duration of symptoms post THC administration (see Supplementary Table 1).

In both studies using orally administration, symptoms peaked at 120 mins for all domains reported (positive, negative, total) and had not fully resolved by the last timepoint reported (120mins and 180mins respectively).

Of 4 studies using inhaled THC, one did not report timings and two used multiple doses of THC, measuring a single time point per dose administered. In the only inhaled study reporting multiple timepoints, total symptoms peaked at 21 minutes and had not fully resolved at 102 minutes.

Finally, of 10 IV studies, symptoms peaked between 10-30 minutes in 8 studies. A single study peaked at 70 minutes although did not report earlier symptom measures. A final study reported mean positive symptoms peaking at 120 mins and negative symptoms at 65 minutes despite also measuring symptoms at 10 minutes, although the timings reported in this study are unclear. Regarding resolution of symptoms, negative and general symptoms resolved at a minimum of 80 minutes, positive symptoms at a minimum of 120 mins, and total symptoms at a minimum of 200 minutes, although total symptoms were not reported in several of the studies reporting earlier timepoints.

Appendix 4. Sensitivity analysis excluding potentially duplicated sample

Since we were unable to confirm that two of the samples included in the positive symptom analysis were independent,(10,11) we performed a sensitivity analysis excluding the study with the smaller sample.(10)

Positive symptoms were thus assessed in 13 studies (14 independent samples) involving a total of 309 participants. THC significantly increased positive symptom severity compared to placebo (SMC = 0.95, 95% CI: 0.72-1.19, p<0.0001). The result remained significant in all iterations of the leave-one-out-analysis (SMC = 0.89-1.01).

There was medium between-sample inconsistency (I^2 = 63.75%, Cochran Q = 40.07, p<0.0001). Egger's test implied significant publication bias (p=0.0001). Trim-fill analysis did not identify any missing studies.

When comparing THC's effects on different symptom domains, THC's effect on positive symptoms remained significantly greater than on negative symptoms (z=2.29, p=0.022) but equivalent to its effect on general symptoms (z=0.23, p=0.82).

Appendix 5. Re-fitting the model comparing symptom domains with $r = 0.1\text{-}0.7$

When comparing positive and negative symptoms induced by the acute administration of THC, there was no significant difference when $r = 0.1$ (z=1.53, p=0.13) but the effect on positive symptoms was significantly greater when $r = 0.5$ (z=2.06, p=0.039) or $r = 0.7$ (z=2.31, p=0.02).

When comparing positive and general symptoms, there was no significant difference at either $r = 0.1$ (z=0.02, p=0.98), $r = 0.5$ (z=0.44, p=0.66) or $r = 0.7$ (z=0.72, p=0.47).

When comparing negative and general symptoms, there was no significant difference when *r* = 0.1 (z=1.70, p=0.09) or *r* = 0.5 (z=1.90, p=0.058) but the effect on general symptoms was significantly greater than negative symptoms when *r* = 0.7 (z=2.01, p=0.044).

Appendix 6. Sensitivity analysis for lower estimation total cannabis exposures

There was no association found between frequent use and positive symptoms (z=1.57, p=0.12), negative symptoms (z=0.79, p=0.43), general symptoms (z=0.97, p=0.33) or total symptoms (z=0.53, p=0.59).

Appendix 7. Sensitivity analysis for higher estimation of total cannabis exposures

There was no association found between frequent use and positive symptoms (z=0.87, p=0.38), negative symptoms (z=0.23, p=0.82), general symptoms (-0.07, p=0.95) or total symptoms (z=-0.35, p=0.73).

Appendix 8. Supplementary analysis including studies using the CAPE scale

We identified two within person studies that had used the CAPE and two between person studies (Supplementary Figure 11.(12,13) All four reported a significant increase in symptoms following administration of THC compared to placebo (Supplementary Table 11)."

Of the within-person studies, one study reported PANSS positive scores in addition to CAPE positive scores and so was already included in the positive symptoms meta-analysis.(12) The second study only reported CAPE negative scores (PANSS negative subscale was also measured but the results are not reported in the text and the author did not respond to requests for data).(13) As there are marked differences between the rating procedure for CAPE relative to the other instruments (the CAPE is a self-reported measure, whereas the PANSS and BPRS are rated by a trained researcher using semi-structured interviews) we excluded the CAPE from our main analysis of negative symptoms. However, we performed a sensitivity analysis including this study for completeness.

Negative symptoms were thus assessed in 13 studies (14 independent samples) involving a total of 286 participants. THC significantly increased negative symptom severity compared to placebo (SMC = 0.75, 95% CI: 0.58-0.93, p<0.0001). The result remained significant in all iterations of the leave-one-out-analysis (SMC = 0.70-0.80).

There was medium between-sample inconsistency (I^2 = 38.52%, Cochran Q = 25.39, p=0.02). Egger's test implied significant publication bias (p=0.0061). Trim-fill analysis identified one putative missing study on the left side. On imputation of the putative missing study, the effect of THC remained highly significant (SMC = 0.73, 95% CI: 0.54-0.91, p<0.0001).

When comparing THC's effects on different symptom domains, THC's effect on positive symptoms remained significantly greater than on negative symptoms (z=2.38, p=0.018). However, its effect on general symptoms became significantly greater when compared to negative symptoms with the additional study included (z=2.14, p=0.032).

Appendix 9. Leave one-out analysis full results

Total symptoms

	estimate	se	zval	pval	ci.lb	ci.ub
Bhattacharyya, 2015	1.1427	0.1118	10.2195	0.0000	0.9235	1.3618
D'Souza, 2012	1.0371	0.0956	10.8491	0.0000	0.8498	1.2245
D'Souza, 2004	1.1300	0.1032	10.9510	0.0000	0.9278	1.3322
D'Souza, 2008	1.0974	0.1000	10.9715	0.0000	0.9013	1.2934
D'Souza, 2009a	1.0771	0.0942	11.4339	0.0000	0.8925	1.2617
D'Souza, 2009b	1.1235	0.0964	11.6548	0.0000	0.9346	1.3124
Radhakrishnan, 2015	1.1257	0.1071	10.5109	0.0000	0.9158	1.3356
Bossong, 2009	1.0931	0.0930	11.7485	0.0000	0.9107	1.2754
Liem-Moolenaar, 2010	1.1050	0.0964	11.4644	0.0000	0.9161	1.2939
Kleinloog, 2012	1.1494	0.1025	11.2132	0.0000	0.9485	1.3502

Positive symptoms

	estimate	se	zval	pval	ci.lb	ci.ub
Barkus, 2011	0.8661	0.1144	7.5736	0.0000	0.6420	1.0902
Bhattacharyya, 2015	0.9342	0.1289	7.2492	0.0000	0.6816	1.1868
D'Souza, 2012	0.8493	0.1133	7.4953	0.0000	0.6273	1.0714
D'Souza, 2004	0.8865	0.1236	7.1720	0.0000	0.6443	1.1288
D'Souza, 2008	0.9021	0.1270	7.1009	0.0000	0.6531	1.1511
D'Souza, 2009a	0.8841	0.1221	7.2430	0.0000	0.6449	1.1234
D'Souza, 2009b	0.9377	0.1237	7.5812	0.0000	0.6953	1.1801
Morrison, 2009	0.9353	0.1272	7.3540	0.0000	0.6861	1.1846
Morrison, 2011	0.8960	0.1252	7.1539	0.0000	0.6505	1.1415
Ranganathan, 2012	0.8935	0.1264	7.0703	0.0000	0.6458	1.1412
Bhattacharyya, 2009	0.9515	0.1198	7.9425	0.0000	0.7167	1.1863
Morgan, 2018	0.9636	0.1084	8.8904	0.0000	0.7512	1.1761
Liem-Moolenaar, 2010	0.9209	0.1264	7.2855	0.0000	0.6732	1.1687
Kleinloog, 2012	0.9251	0.1296	7.1385	0.0000	0.6711	1.1792
Radhakrishnan, 2015	0.8809	0.1230	7.1601	0.0000	0.6397	1.1220

Negative symptoms

	estimate	se	zval	pval	ci.lb	ci.ub
Barkus, 2011	0.7444	0.0911	8.1703	0.0000	0.5658	0.9230
D'Souza, 2012	0.7236	0.0896	8.0772	0.0000	0.5480	0.8991
D'Souza, 2004	0.7791	0.1046	7.4463	0.0000	0.5740	0.9842
D'Souza, 2008	0.7653	0.1022	7.4856	0.0000	0.5649	0.9657
D'Souza, 2009a	0.8044	0.1034	7.7815	0.0000	0.6018	1.0070
D'Souza, 2009b	0.8023	0.1015	7.9055	0.0000	0.6034	1.0013
Morrison, 2011	0.7579	0.0990	7.6571	0.0000	0.5639	0.9519
Ranganathan, 2012	0.7743	0.1057	7.3258	0.0000	0.5671	0.9815
Bhattacharyya, 2012	0.7650	0.1007	7.5985	0.0000	0.5677	0.9624
Morgan, 2018	0.8305	0.0868	9.5637	0.0000	0.6603	1.0007
Liem-Moolenaar, 2010	0.8164	0.0969	8.4274	0.0000	0.6265	1.0063
Kleinloog, 2012	0.7932	0.1089	7.2863	0.0000	0.5798	1.0065
Radhakrishnan, 2015	0.8003	0.1068	7.4945	0.0000	0.5910	1.0096

General symptoms

	estimate	se	zval	pval	ci.lb	ci.ub
Barkus, 2011	0.9662	0.1124	8.5991	0.0000	0.7459	1.1864
D'Souza, 2012	0.9020	0.1050	8.5928	0.0000	0.6963	1.1077
D'Souza, 2004	1.0724	0.1422	7.5409	0.0000	0.7937	1.3512
D'Souza, 2008	1.0586	0.1500	7.0557	0.0000	0.7645	1.3526
D'Souza, 2009a	0.9660	0.1212	7.9721	0.0000	0.7285	1.2036
D'Souza, 2009b	1.0402	0.1396	7.4513	0.0000	0.7666	1.3138
Liem-Moolenaar, 2010	0.9850	0.1268	7.7707	0.0000	0.7366	1.2335
Kleinloog, 2012	1.0746	0.1522	7.0611	0.0000	0.7763	1.3729
Radhakrishnan, 2015	1.0789	0.1381	7.8114	0.0000	0.8082	1.3496

References

1. Web Plot Digitizer [Internet]. [cited 2019 Jul 4]. Available from: https://automeris.io/WebPlotDigitizer/

2. Karcher NR, Barch DM, Demers CH, Baranger DAA, Heath AC, Lynskey MT, et al. Genetic Predisposition vs Individual-Specific Processes in the Association Between Psychotic-like Experiences and Cannabis UsePredisposition vs Individual Processes in Psychotic-like Experiences in Cannabis UsersPredisposition vs Individual Processes in P. JAMA Psychiatry [Internet]. 2019 Jan 1;76(1):87–94. Available from: https://doi.org/10.1001/jamapsychiatry.2018.2546

3. Kasten CR, Zhang Y, Boehm 2nd SL. Acute and long-term effects of Δ9-tetrahydrocannabinol on object recognition and anxiety-like activity are age- and strain-dependent in mice. Pharmacol Biochem Behav [Internet]. 2017/10/28. 2017 Dec;163:9–19. Available from: https://www.ncbi.nlm.nih.gov/pubmed/29107728

4. Normandin MD, Zheng M-Q, Lin K-S, Mason NS, Lin S-F, Ropchan J, et al. Imaging the cannabinoid CB1 receptor in humans with [11C] OMAR: assessment of kinetic analysis methods, test–retest reproducibility, and gender differences. J Cereb Blood Flow Metab. 2015;35(8):1313–22.

5. Hirvonen J, Zanotti-Fregonara P, Gorelick DA, Lyoo CH, Rallis-Frutos D, Morse C, et al. Decreased Cannabinoid CB1 Receptors in Male Tobacco Smokers Examined With Positron Emission Tomography. Biol Psychiatry [Internet]. 2018;84(10):715–21. Available from: http://www.sciencedirect.com/science/article/pii/S0006322318316792

6. Karcher NR, Barch DM, Demers CH, Baranger DAA, Heath AC, Lynskey MT, et al. Genetic Predisposition vs Individual-Specific Processes in the Association between Psychotic-like Experiences and Cannabis Use. JAMA Psychiatry. 2019 Jan;76(1):87–94.

7. Ohlsson A, Lindgren J-E, Wahlen A, Agurell S, Hollister LE, Gillespie HK. Plasma delta-9-tetrahydrocannabinol concentrations and clinical effects after oral and intravenous administration and smoking. Clin Pharmacol Ther [Internet]. 1980 Sep 1;28(3):409–16. Available from: https://doi.org/10.1038/clpt.1980.181

8. Bell M, Milstein R, Beam-Goulet J, Lysaker P, Cicchetti D. The positive and negative syndrome scale and the brief psychiatric rating scale: Reliability, comparability, and predictive validity. J Nerv Ment Dis. 1992;

9. Brugger SP, Howes OD. Heterogeneity and homogeneity of regional brain structure in schizophrenia: a meta-analysis. JAMA psychiatry. 2017;74(11):1104–11.

10. Bhattacharyya S, Fusar-Poli P, Borgwardt S, Martin-Santos R, Nosarti C, O'Carroll C, et al. Modulation of Mediotemporal and Ventrostriatal Function in Humans by Δ9-Tetrahydrocannabinol. Arch Gen Psychiatry. 2009;66(4):442.

11. Bhattacharyya S, Atakan Z, Martin-Santos R, Crippa JA, Kambeitz J, Malhi S, et al. Impairment of inhibitory control processing related to acute psychotomimetic effects of cannabis. Eur Neuropsychopharmacol [Internet]. 2015;25(1):26–37. Available from: http://dx.doi.org/10.1016/j.euroneuro.2014.11.018

12. Morrison PD, Zois V, McKeown DA, Lee TD, Holt DW, Powell JF, et al. The acute effects of synthetic intravenous 9- tetrahydrocannabinol on psychosis, mood and cognitive functioning. Psychol Med. 2009;39(10):1607–16.

13. Morrison PD, Stone JM. Synthetic delta-9-tetrahydrocannabinol elicits schizophrenia-like negative symptoms which are distinct from sedation. Hum Psychopharmacol Clin Exp. 2011;26(1):77–80.
